# The Effect of Degree of Milling on the Nutraceutical Content in Ecofriendly and Conventional Rice (*Oryza sativa* L.)

**DOI:** 10.3390/foods9091297

**Published:** 2020-09-15

**Authors:** Seung-Hyun Kim, Yu-Jin Yang, Ill-Min Chung

**Affiliations:** Department of Crop Science, College of Sanghuh Life Science, Konkuk University, 120 Neungdong-ro, Gwangjin-gu, Seoul 05029, Korea; kshkim@konkuk.ac.kr (S.-H.K.); jin0931@konkuk.ac.kr (Y.-J.Y.)

**Keywords:** ecofriendly rice, degree of milling, nutraceuticals, DPPH radical scavenging activity, discriminant analysis

## Abstract

We investigated the effects of the type of rice and degree of milling (DOM) on the nutraceutical content and antioxidant activity of rice (*Oryza sativa* L.). The fatty acid (FA), vitamin E homolog, and phenolic contents in organic (OR), pesticide-free (PFR), and conventional rice (CR) decreased significantly with an increase in the DOM of rice grains, particularly for a DOM of 7 and 9 (*p* < 0.05). The 2,2-diphenyl-1-picrylhydrazyl (DPPH) radical scavenging activity also decreased with the DOM; particularly, this activity decreased significantly, by approximately 60%, in rice grains with a DOM between 7 and 11, as compared to that of brown rice (*p* < 0.05). α-Tocopherol (*r* = 0.854) and *p*-coumaric acid (*r* = 0.501) showed the strongest correlation with DPPH activity in each chemical group. Stepwise discriminant analysis enabled the correct original and cross-validated classification of 87.0% and 81.5% of rice types, respectively. Additionally, the original and cross-validated classification of rice DOM levels showed that, overall, 93.8% and 92.6% of rice samples were correctly classified. Our findings reveal variations in the nutraceutical levels and antioxidant activities in rice grains based on the rice type and DOM, which can help improve the nutritional evaluation of human-health-promoting rice grains.

## 1. Introduction

Rice (*Oryza sativa* L.) is a staple food for nearly half of the world’s population, especially in most Asian countries. Rice grains contain many nutrient components, including carbohydrates, proteins, crude fat, dietary fiber, and micronutrients such as vitamins and minerals [1]. Additionally, they contain essential amino acids and many bioactive non-nutrient compounds, such as phenolic compounds [2]. Specifically, fatty acid (FA), vitamin E (VitE), and certain phenolics found in rice grains have been widely reported to be involved in several human-health-related functions such as energy metabolism; decreased chronic disease risk; and antioxidant, anti-inflammatory, anti-carcinogenic, and anti-cardiovascular activities [3,4,5].

A rice grain consists of the husk, bran (pericarp, seed coat, and aleurone layer), embryo, and inner endosperm. The process of milling strips away the bran layer from the grain. Rice can be classified as either brown rice (BR) or polished rice based on the degree of milling (DOM). BR is dehulled to remove the husk alone; hence, it contains an embryo, endosperm, and bran. If the bran layer is stripped away, the grain is classified as diverse polished rice according to the DOM. A DOM of rice between 5 and 11 indicates the retention of 50% and 5% of the embryo and bran layer, respectively, for BR. Additionally, rice with a DOM of 13 typically does not contain an embryo or an aleurone layer [6]. Rice with a DOM >10 is generally referred to as white (polished) rice. In the past, BR was not widely consumed because of its harsh texture and low digestibility. However, the consumption of BR has consistently increased with increasing interest in health by consumers. The bioactive components of rice widely differ depending on the DOM. Many nutraceuticals are present in BR in addition to starch, and certain chemical constituents other than starch are concentrated in the pericarp, seed coat, and aleurone layer of BR [7]. These components are removed during the milling process; therefore, the intake of nutraceuticals present in rice grain can widely differ with the DOM.

Many people have recently preferred to consume organic agricultural products, including organically cultivated BR, because of an increased awareness of health and environmental problems. In Korea, the National Agricultural Products Quality Management Service (NAQS) provides certificates for agricultural products that are organic or pesticide-free as ecofriendly. The ecofriendly label means that either no or minimal synthetic pesticides, chemical fertilizers, antibiotics, bactericides, and other chemical materials are used and that the byproducts of farming, animal husbandry, and forestry are recycled. Ecofriendly approaches aim to maintain the farming ecosystem and preserve the environment during the production of agricultural products, including livestock products. Specifically, products cultivated without using synthetic agricultural pesticides or chemical fertilizers are known as organic products. Products cultivated without the use of synthetic agricultural pesticides and <1/3rd of the recommended fertilizer amount are known as pesticide-free products. Finally, conventional agricultural products are not certified and are typically cultivated using agricultural products, including organosynthetic agricultural pesticides or chemical fertilizers.

Although it remains controversial, organic or pesticide-free products are generally considered as safer and healthier than conventional products; it has been predicted that because the defense mechanisms in plants are decreased due to the use of chemically synthesized pesticides or fertilizers, the levels of minerals, vitamins, and secondary metabolites produced may also be decreased [8]. Furthermore, organic agricultural products may contain more nutrients because they can absorb more mineral and microelements via the sufficient activity of soil microbes [9]. According to some previous studies, organic rice (OR) had slightly higher amylose, protein, and fiber contents than conventional rice (CR) [8,10]. Additionally, OR exhibits higher total phenolic, phytic acid, and mineral contents than CR. However, the effects of the organically cultivated paddy-rice system and its various health-promoting activities, bioactive components, and their correlation have been insufficiently explored compared to those of the conventional production system [10].

It is important to set standards for discriminating between OR, pesticide-free rice (PFR), and CR and increase the consumption of organic agricultural products; therefore, studies on the differences in chemical contents and bioactivities of different rice types are necessary. Furthermore, few studies have examined nutraceuticals and bioactivity while considering the rice DOM and type simultaneously. Therefore, we examined the differences in the nutraceutical content in OR, PFR, and CR depending on the DOM and validated the antioxidative activity, which depends on the DPPH free radical scavenging activity.

## 2. Materials and Methods

### 2.1. Preparation of Polished Rice Samples

**Figure 1:**
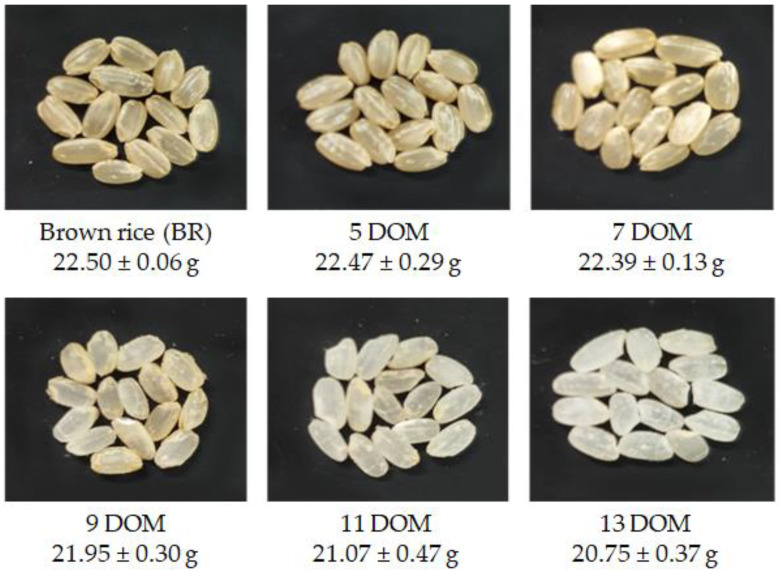
Representative photos and the 1000-grain weight (g, mean ± SD) of rice, according to degree of milling (DOM) (brown rice (BR) to 13 DOM). The 1000-grain weight is a factor of the weight of grains and the number of grains (1000), which is one of the important agronomic traits. Here, the 1000-grain weight shows clearly how much the rice grain weight varied depending on rice milling degree.

A total nine BR samples, which consisted of three brands per OR, PFR, and CR, was purchased from several retail markets in Korea in 2019. We used a mixed rice sample obtained from common retail markets, in which the rice cultivars were not specified. All BR samples at five levels of DOMs (i.e., 5, 7, 9, 11, and 13 DOM) were polished using a rice polishing machine (NRH-500, Narok Co., Ltd., Hwaseong-si, Korea). Each DOM is defined in terms of the proportion of embryo and bran layer remaining in the whole rice grain [6]. Therefore, BR, 5, 7, 9, 11, and 13 DOM indicate that 100%, 50%, 30%, 15%, 5%, and 0% of the embryo and bran layer in the rice grain are retained. Thereafter, the samples were lyophilized at <−45 °C for 3 days (Freexeone 4.5, Labconco, Kansas City, MO, USA) and ground to generate powdered samples with particle sizes of <400 µm. All rice samples were temporarily stored in a desiccator (room temperature, humidity: <15–20%) until analysis and sample preparation could be performed. Samples kept for more than 1 week were stored at −70 °C (Figure 1).

### 2.2. Chemicals and Reagents

All chemicals used for the extraction and analysis of FAs, VitE, and phenolic compounds were of the HPLC-grade or at least ACS analytical grade. Acetonitrile, ethanol, methanol (MeOH), and iso-octane were obtained from Thermo Fisher Scientific (Waltham, MA, USA). Deionized water was generated using the PURELAB Option-Q System (ELGA Lab Water, High Wycombe, UK). Potassium hydroxide, benzene, formic acid, and heptane were purchased from Junsei (Tokyo, Japan). Hexane was purchased from Kanto Chemical Co., Inc. (Tokyo, Japan). Dichloromethane, 2,2-dimethoxypropane, and dimethyl sulfoxide were purchased from Sigma-Aldrich Corp (St. Louis, MO, USA). Sulfuric acid, sodium sulfate anhydrous, and 0.1 N hydrochloric acid were purchased from Daejung Chemical and Materials Co., Ltd. (Gyeonggi-Do, Korea). Ascorbic acid was obtained from SANCHUN Chemical Co., Ltd. (Gyeonggi-Do, Korea). The 37 fatty acid methyl ester (FAME) standard mixture, pentadecanoic acid (C15:0, internal standard), VitE (4 tocopherols and 4 tocotrienols), and phenolic standards (STDs) were phurchased from Sigma-Aldrich Co. We obtained 2,2-diphenyl-1-picrylhydrazyl (DPPH) from Alfa Aesar (Ward Hill, MA, USA).

### 2.3. Sample Extraction, Analysis, and Quantification

#### 2.3.1. Extraction and Conversion of FAME

FA extraction and FAME conversion were simultaneously conducted as previously described [11]. Transesterification in rice samples was performed to convert FA to FAME before using a gas chromatography apparatus comprised of a flame ionization detector (GC-FID) for analysis. Rice powder sample (50 mg) was added into an amber vial, after which C15:0 (0.2 mg) was added to the same vial as the internal standard (IS). Thereafter, heptane (400 μL) and the methylation mixture solution (680 μL) were added to the samples in the vial. The methylation mixture consisted of MeOH: benzene: 2,2-dimethoxypropane: sulfuric acid in a ratio of 19.5:10:2.5:1 (*v/v/v/v*). The vials were placed in a water bath/shaker (BF-45SB, Biofree Co. Ltd., Cheongju-Si, Korea) at 40 rpm and 80 °C, for 2 h. The samples were then cooled for 30 min, and the supernatants were collected in a microcentrifuge tube. The collected supernatants were further centrifuged at approximately 45× *g* for 1 min. Finally, the supernatant in the microcentrifuge tube was transferred to a 300-μL-sized insert in the amber vial for analysis by GC-FID (Agilent 7890 B, Agilent Technologies, Santa Clara, CA, USA).

#### 2.3.2. FAME Analysis by GC-FID

FAME analysis was conducted using GC-FID, along with a capillary column (HP-INNOWAX N, 0.25 mm × 30 m, 0.25 μm, Agilent Technologies). The injection volume was set at 1 μL in 1:50 split mode. Helium was used as the carrier gas at a flow rate of 10 mL·min^−1^. The hydrogen as the flame gas was flowed at a rate of 35 mL·min^−1^, whereas the mixed gas (air gas) was flowed at a rate of 300 mL/min. The oven temperature was initially set to 100 °C for 2 min, after which it was increased to 150 °C (5 °C·min^−1^) over 2 min, and then increased to 240 °C (5 °C·min^−1^) for 5 min. The inlet and FID detector temperatures were set to 230 and 250 °C, respectively. The total analytical time period was 64 min per sample [12]. Representative chromatograms of the 37 FAME STD mixture and samples of interest are shown in Figure S1. One milliliter of the mixture containing 37 FAMEs was dissolved in 9 mL of dichloromethane. FAs in the samples were identified by comparing their retention times with that of the standard FAME mixture. Additionally, a FAME STD was added to the same samples to obtain peaks; this is commonly known as a ‘spiking test’. The FA content of the samples was calculated as described by the Ministry of Food and Drug Safety in Korea

$$\mathrm{Fatty}\;\mathrm{Acid}\;(\mathrm{mg}\cdot g^{-1})\;=\;\frac{P_{ti\;}\times F_{i}\times F_{IS}\times Wt_{IS}}{Pt_{IS}\times R_{i}\times W_{spl}}\times 100,\;R_{i}\;=\;\frac{Ps_{i}}{Ps_{IS}}\times \frac{W_{IS}}{W_{i}}$$

Here, *Pt_i_* is the peak area of FA *i*, *Pt_IS_* is the peak area of the internal standard (IS), *F_i_* is the conversion factor of the FA *i*, F_IS_ is the conversion factor of the IS, *Wt_IS_* is the amount of IS, *W_spl_* is the amount of sample of interest, R_i_ is the response factor of FA *i*, *Ps_i_* is the peak area of FA *i* in the standard mixture, *Ps_IS_* is the peak area of IS in the standard mixture, *W_IS_* is the amount of IS in the standard mixture, and *W_i_* is the amount of FA *i* in the standard mixture.

#### 2.3.3. VitE Extraction

VitE was extracted as previously described [12]. One gram of each rice sample containing 0.1 g of ascorbic acid was extracted with 20 mL of ethanol and placed in a water bath/shaker at 80 °C and 160 rpm for 10 min. Saponification was performed by adding 300 μL of saturated potassium hydroxide; the contents were then placed in a water bath/shaker at 80 °C and 160 rpm for 18 min. After cooling, 10 mL of hexane and 10 mL of water were added to the sample. After shaking, the samples were centrifuged at 3000× *g* at 4 °C for 5 min. The upper layer was collected into another conical tube. We added 10 mL of hexane to the sample remnants and centrifuged the tubes again. This procedure was performed three times, giving 30 mL of the hexane layer. Next, 30 mL of hexane was filtered through sodium sulfate anhydrous. Finally, the aliquot was concentrated using a rotary vacuum evaporator (EYELA SB-1200, Tokyo Rikakikai Co., Ltd., Tokyo, Japan). The residue was dissolved in 1 mL of iso-octane and the contents were transferred to an amber vial.

#### 2.3.4. VitE Analysis by GC-FID

VitE analysis was conducted by performing GC-FID along using a capillary column (CP-SIL 8 CB, 0.32 mm × 50 m, 0.25 μm). The injection volume was set to 1 μL in 1:20 split mode. The carrier gas, N_2_, was set to 25 mL·min^−1^. Hydrogen gas and air were set to 30 and 400 mL·min^−1^, respectively, for the FID flame. The initial oven temperature was set to 220 °C for 2 min, and then increased to 290 °C (5 °C min^−1^), which was maintained for 14 min. Next, the temperature was increased to 300 °C (10 °C min^−1^), and maintained for 10 min. Both the inlet and FID temperatures were set to 290 °C. The tocopherol (αT, βT, γT, δT) and tocotrienol (αT_3_, βT_3_, γT_3_, δT_3_) STDs were dissolved in isooctane to prepare stock solutions. VitE in the samples was identified by comparing the retention time with that of the authentic mixture of VitE STDs. Additionally, a VitE STD was used to spike the same samples to obtain and validate peaks with certain values. All VitE calibration curves exhibited good linearity (r^2^ > 0.99); the limit of detection (LOD = 3 × SD/S) and limit of quantitation (LOQ = 10 × SD/S) were calculated from the prepared calibration curves, where SD was the standard deviation of the y-intercept of the calibration curve and S was the slope of each calibration curve [12]. Representative chromatograms of the VitE STDs and samples of interest are shown in Figure S2.

#### 2.3.5. Extraction of Phenolic Compounds

Phenolic compounds were extracted using the previously described acidic extraction method [13]. One gram of rice sample was placed in an Erlenmeyer flask and extracted with 10 mL of acetonitrile and 2 mL of 0.1 N hydrochloric acid. The sample was placed in a shaker (Green-SSeriker, Vision Scientific Co., Ltd., Gyeonggi-Do, Korea) at 200 rpm and approximately 25 °C (room temperature) for 2 h. The extracted sample was filtered through Whatman No.42 filter paper (GE Healthcare Life Science, Little Chalfont, UK). The extracted aliquot was collected in a round flask and concentrated at below 33 °C i a rotary vacuum evaporator. The resulting residue was dissolved in 5 mL of 80% MeOH and filtered through a PTFE membrane syringe filter (13 mm diameter, 0.22 µm, Thermo Fisher Scientific).

#### 2.3.6. Phenolic Compound Analysis by LC-MS/MS

Phenolic compounds were analyzed by high-performance liquid chromatography (HPLC)–electrospray ionization (ESI)–tandem mass spectrometry (triple quadrupole); the analytical conditions have been reported previously [13]. The mobile phase consisted of solvent A, which contained 0.1% formic acid in deionized water, and solvent B, which contained 0.1% formic acid in acetonitrile. The mobile phase gradient was used for separation over certain time periods, as follows: 0 min, 90% A; 10 min, 60% A; 20 min, 50% A; 25 min, 0% A, 26–30 min, 90% A for re-equilibrium. The injection volume was 10 μL and the mobile phase flow rate was 0.5 mL/min. Phenolics in the samples were separated using a reversed-phase column (C18 Thermo Syncronicsm, 150 × 4.6 mm, 5 µm).

We used negative ESI mode and multiple reaction monitoring (MRM) to measure 54 selected phenolics, after setting the following parameters: collision N_2_ (2 psi), curtain gas (50 psi), nebulizer N_2_ (40 psi), heater N_2_ (50 psi), ion-spray voltage (−4400 V), and probe temperature (500 °C). The ion spray probe was located at 5 and 3 mm on the horizontal and vertical axes, respectively. The MRM parameters of the 54 selected phenolics were optimized by infusing the corresponding phenolic STDs at concentrations of 10–20 ppm (Table S1).

Phenolics in the samples of interest were identified by comparing their retention times and mass values to the charge ratio values (*m/z*) of equivalent phenolic STDs. The phenolic STDs were dissolved in adequate solvents, and their solubility-related properties were used to prepare stock solutions. To construct each calibration curve, the STD stock solution was diluted with 80% MeOH, taking into account the phenolic content present in the samples of interest. The LOD (3:1) and LOQ (10:1) were determined from the signal and noise (S/N) ratio, representative MRM ion chromatograms, and spectra of the phenolic STD mixture; the sample measurement is shown in Table S2 and Figure S3.

#### 2.3.7. DPPH Free Radical Scavenging Activity

The DPPH free radical scavenging activities of the rice samples of interest were measured as previously described [14]. The rice (0.1 g) was extracted in 2 mL of 80% MeOH and placed in a shaker at 300 rpm for 60 min. The sample extract was centrifuged at 3000× *g* for 3 min at approximately 25 °C, and the supernatant was used for DPPH activity measurement. An extract solution was prepared; 1.9 mL of DPPH solution (0.1 mM in methanol) was transferred to a cuvette (10 × 10 × 45 mm, 4 mL, Ratiolab GmbH disposables for sciences, Dreieich, Germany) along with 0.1 mL rice extract. This mixture was placed in the dark for 30 min. Finally, the absorbance was measured using an OPTIZEN POP UV-spectrophotometer (Mecasys Co., Daejeon, Korea) at 517 nm. Antioxidative activity was calculated as follows: % DPPH radical scavenging activity = ((Absorbance_control_ − Absrobance_sample_)/Absorbance_control_) × 100.

### 2.4. Statistical Analysis

In this study, all sample extractions and analyses were conducted in triplicate. Statistical analysis of replicated data was performed using SAS software (version 9.4; SAS Institute, Inc., Cary, NC, USA). A general linear model was prepared, and the least significant difference test was conducted at the 0.05 probability level. Two-way analysis of variance was conducted for the different degrees of milling and rice types. Further, a hierarchical correlation analysis was conducted between phenolic compounds, VitE, and DPPH free radical scavenging activity using SAS and the online Morpheus software (Morpheus, https://software.broadinstitute.org/morpheus). Moreover, all data obtained by measuring FA, vitamin E, and phenolic levels were subjected to discriminant analysis (DA) to develop a predictive model for each rice type and rice DOM identification (SPSS version 24, SPSS, Inc., Chicago, IL, USA). Here, for the equal-sized grouping of variables (three rice types and six rice DOM levels), all measured independent values used during stepwise DA (SDA), along with Wilks’s Lamda method. Cross-validation was performed for unknown group membership using the leave-one-out classification method; the results were evaluated to determine the accuracy of the group membership classification.

## 3. Results and Discussion

### 3.1. Effect of DOM on Chemical Composition and Content of Rice

#### 3.1.1. FA Variation in Rice Grains According to the DOM

**Figure 2:**
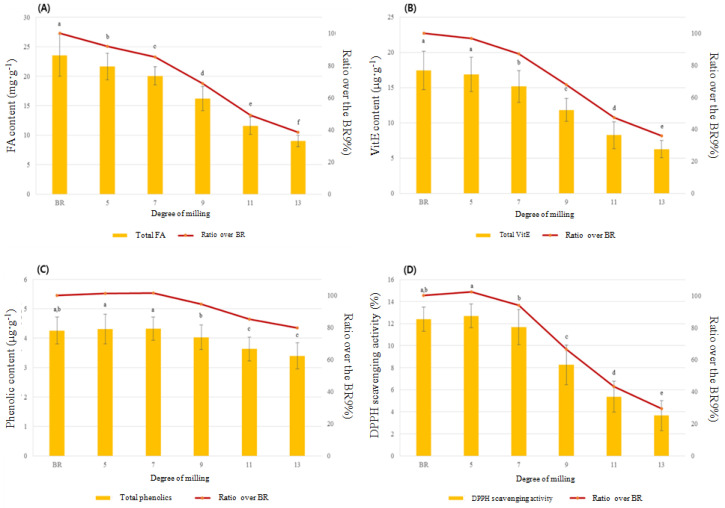
Comparison of (**A**) total fatty acid (FA), (**B**) total vitamin E (VitE), (**C**) total phenolic content and proportion, and (**D**) 2,2-diphenyl-1-picrylhydrazyl (DPPH) free radical scavenging activity according to the DOM. Different superscripts ^(a–f)^ represent a significant difference, depending on the DOM (*p* < 0.05).

**Figure 3:**
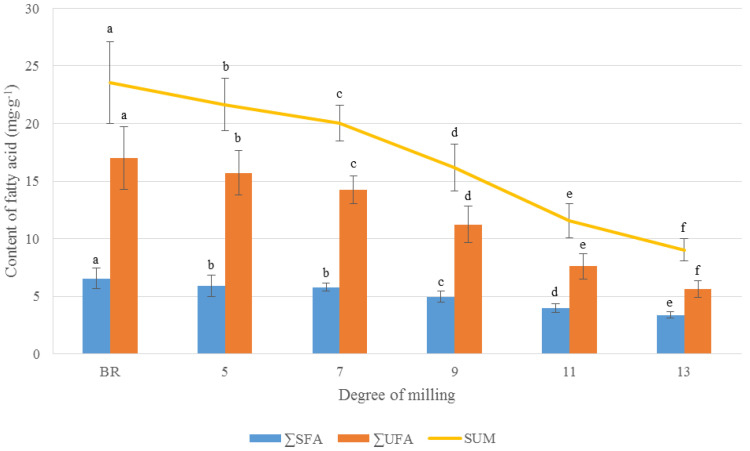
Comparison of the total FA, unsaturated (U)FA, and saturated (S)FA content of rice grains according to the DOM. Different superscripts ^(a–f)^ represent a significant difference in the FA content of rice, depending on the DOM (*p* < 0.05).

The total FA content was decreased by an average of 14.52 mg·g^−1^ as DOM increased from BR to 13 DOM, accounting for approximately 62% of the total FA content in BR. Particularly, the total FA content was greatly decreased between 7 and 11 DOM by around 36% (Figure 2A). The unsaturated FA (UFA) content was greatly decreased by approximately 11 mg·g^−1^ between BR and 13 DOM rice, whereas the saturated FA (SFA) content was decreased by around 3 mg·g^−1^ from BR to 13 DOM. Interestingly, the proportion of SFAs among the total FA content in 13 DOM rice appeared to be approximately 10%, which was an increase compared to that in BR, despite the decrease in the absolute amount of SFA. The proportion of UFA compared to the total FA content of rice was decreased by 10% between BR and 13 DOM rice (Figure 3).

In addition, the levels of ten FAs in all rice samples significantly differed among the rice grains according to the DOM (*p* < 0.0001, Table S3). Linoleic acid (C18:2n6), oleic acid (C18:1n9), and palmitic acid (C16:0) were the most abundant FAs in all DOM rice samples, accounting for approximately 94% of the total FAs. Interestingly, the proportion of C18:2n6 and C18:1n9 to the total FAs was decreased by around 9% between BR and 13 DOM rice; however, there was a 10% increase in the C16:0 proportion between BR and 13 DOM rice (Table S3). Additionally, the C18:2n6 and C18:1n9 values significantly decreased as compared to values for 7 DOM rice, whereas the proportion of C16:0 was significantly decreased compared to that of 11 DOM rice. According to a previous report [15], BR with a bran layer contained larger amounts of C18:2n6 and C18:3n3 than did white (polished) rice; however, the ratio of C16:0 to total FA content was higher in white rice. Consequently, the change in FA content and proportion according to the DOM was significantly associated with a decreased UFA content in rice bran. Therefore, BR can be considered as more desirable for consumption because of its large amounts of total FA and larger proportion of UFA, which included essential FAs; these molecules are well known to have human-health-promoting effects such as reduced cholesterol levels.

#### 3.1.2. VitE Variations in Rice Grains According to DOM

The total VitE content was decreased by 64% between BR and 13 DOM rice; specifically, the VitE content in 9 and 11 DOM rice samples was significantly decreased by 32% and 53%, respectively, relative to that in BR (Figure 2B). Among the eight VitE homologs observed in this study, four (i.e., αT, γT, αT_3_, γT_3_) were detected in all samples, and their content was significantly decreased with an increase in the DOM of the rice grain, from that of BR to 13 DOM rice (*p* < 0.001, Table S4). αT and γT_3_ were the major VitE types in all rice samples, accounting for 74–79% of the total VitE content in the rice samples at all DOMs. Compared to the total VitE content in BR, the αT content was decreased by 75% in 13 DOM rice, whereas the γT_3_ content was decreased by 43% in 13 DOM rice. Thus, αT is more abundant in the rice bran layer than the other VitE homologs (Table S4). It is well-known that in its natural form, αT exhibits acceptable levels of antioxidant activity. Additionally, tocotrienols are known to have stronger antioxidant activity than tocopherols because their structures contain an unsaturated chain [16]. Therefore, because of its larger VitE content, especially αT and γT_3_, BR may be associated with greater health-promoting effects than polished rice.

#### 3.1.3. Phenolic Variations in Rice Grains According to DOM

The DOM also affected the phenolic content of rice grains (*p* < 0.0001, Table S5, Figure 2C). The sums of the phenolics measured in this study (same as total phenolic content) were in the following order: 7 DOM, 5 DOM, BR > 9 DOM > 11 DOM, 13 DOM. The total phenolic content was significantly decreased by 5% in 9 DOM rice and 15% in 11 DOM rice as compared to that in BR. However, the maximum difference in the total phenolic content among the different rice DOMs was 0.92 µg·g^−1^ between 7 DOM and 13 DOM rice samples, showing small difference (Table S5, Figure 2C).

Among the 54 selected phenolics measured, nine phenolic compounds, i.e., protocatechuic acid, *p*-hydroxybenzoic acid, gentisic acid, *p*-coumaric acid, salicylic acid, caffeic acid, ferulic acid, veratric acid, and vanillin, were found in all DOM rice samples; however, the levels of two phenolics, i.e., veratric acid and vanillin, appeared to be below the LOD or LOQ (Table S5).

**Figure 4:**
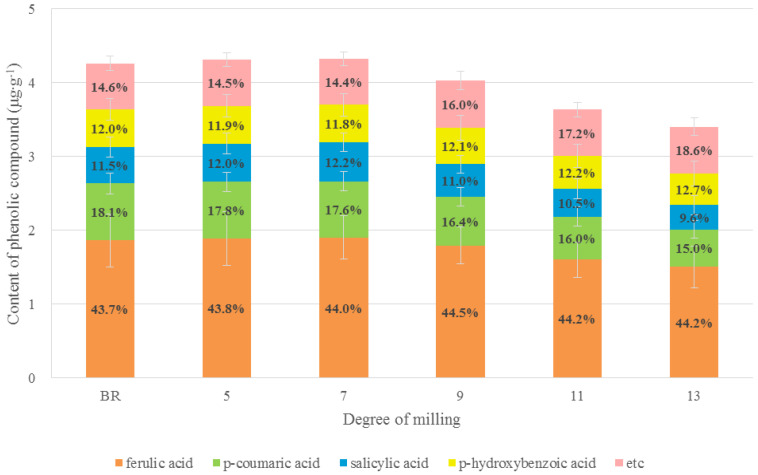
Comparison and variation of phenolic compounds in rice grains according to the DOM.

Ferulic acid was the most abundant phenolic compound in all rice samples, accounting for about 44% of the total phenolic content; its proportion did not differ according to the DOM. *p*-Coumaric, *p*-hydrobenzoic, and salicylic acids appeared to be the next most abundant phenolics in all DOM rice samples, accounting for approximately 10–18% of the total phenolic content. Unlike *p*-coumaric and salicylic acids (*p* < 0.001), *p*-hydroxybenzoic acid levels did not differ with the DOM. Interestingly, the sum of the remaining phenolics (i.e., caffeic, protocatechuic acid, and gentisic acids) in the rice grain was increased by around 4% as the DOM increased (Table S5, Figure 4).

The rice bran and husk contained larger amounts of phenolic acids compared to the endosperm [17], and ferulic and *p*-coumaric acids are the main phenolics in rice grains [18]. Ferulic acid was particularly abundant in the aleurone, pericarp, and embryo cell walls of various grains [19]. Therefore, performing milling to eliminate the rice bran layer strongly affects the phenolic composition and content of rice grains. A previous study [17] reported that phenolic acids were present in BR at higher levels than in polished rice. Additionally, other studies showed that the pericarp, aleurone layer, and embryo fractions of whole BR contained 13%, 28.5% and 8.8% of the phenolics, respectively, whereas the endosperm fraction contained the remaining 49.7% of the total phenolic acid content in BR. Thus, phenolics are more abundant in whole BR than in polished rice, depending on the DOM [20,21]. Specifically, coumaric and ferulic acids, which are typically connected to sugar residues or side chains of xylan polysaccharides in the cell wall through ester linkages, were found to be the main phenolics present in rice bran [22]. Our findings are similar to those of previous studies describing the differences in the phenolic composition and content of rice grains. Subsequently, because nutraceuticals in whole rice grains are mostly found in the inner and outer layers of whole BR in the free, soluble-conjugated, and bound forms, BR can be regarded as a better dietary component than polished rice, a common human dietary component in many countries.

### 3.2. Effect of Rice Type (Organic, Pesticide-Free, Conventional Rice) on Chemical Composition and Rice Grain Content

#### 3.2.1. Comparison of FA Content in Rice Types with the Same DOM

**Figure 5:**
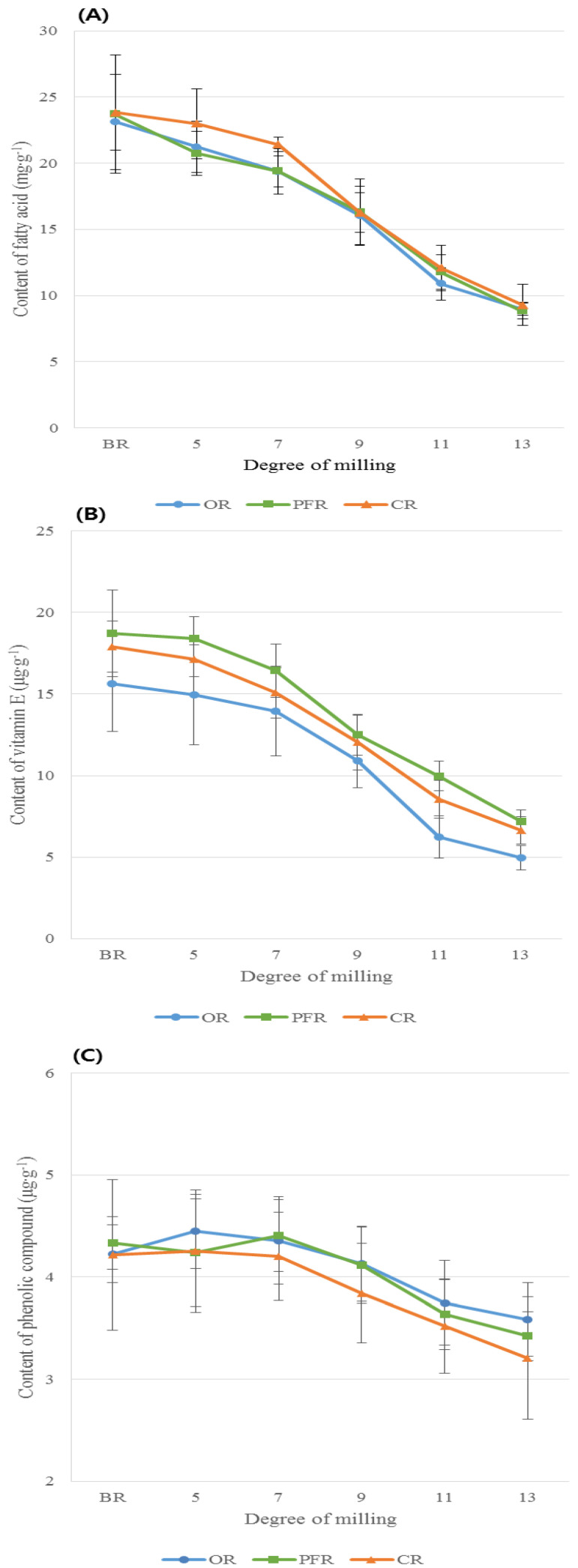
Comparison of (**A**) total fatty acid, (**B**) total vitamin E, and (**C**) total phenolic compound levels by rice type depending on the degree of milling.

Figure 5A shows the total FA content in rice types with the same DOM. All rice types (i.e., OR, PFR, CR) showed decreasing values with an increasing DOM; in particular, significantly decreased CR samples had a DOM of 7–9. However, although the values for rice samples with a DOM of 5 and 7 significantly differed for different rice types, a higher total FA content was observed in CR than in OR or PFR (*p* < 0.05, Table S6, Figure 5A). Among the main FAs (i.e., C16:0, C18:1n9, C18:2n6) measured in this study, only the values for C18:1n9 significantly differed between CR and OR/PFR in the 7, 11, and 13 DOM rice samples. The amounts of other FAs, such as *cis*-11-eicosenoic acid (C20:1n9) and lignoceric acid (C24:0), significantly differed among rice types with the same DOM. However, as these FAs were present in very small quantities, they do not result in strong human health benefits. Additionally, the loss of FAs during the milling process was higher in all rice types with a DOM between 7 and 11; the C18:1n9 level was especially decreased by a maximum of approximately 60% in 11 DOM rice compared to that in BR (Table S6). At all DOM levels, OR and PFR had a relatively lower MUFA/PUFA ratio than CR; the n6/3 ratio was higher in OR and PFR than in CR. This indicates that OR and PFR had greater levels of PUFA, specifically more n6 FAs. The DOM and rice type did not appear to interact in all FAs measured in this study (Table S3).

Few reports have described the comparisons of the differences in FA levels of organic and conventional rice grains. In general, however, crops produced using organic cultivation systems have higher levels of valued nutrients, including secondary metabolites with antioxidant activity, carotenoids, vitamins, total flavonoids, and phenolic acids, than those produced conventionally [23]. Additionally, according to previous studies, a ratio of intake of n6/n3 of 20:1 or more in Western diets resulted in increased pathogenesis of many diseases, including cardiovascular disease, cancer, inflammatory and autoimmune diseases, and an increased risk of obesity, over the last three decades [24,25]. Based on our results, OR and PFR can be considered as good dietary sources, as they represent a higher source of essential FAs than CR at all DOM levels. However, the intake of n6 FAs should be higher than that of n3 FAs because of the adverse health effects described above. Thus, a balanced intake of an n6/n3 ratio that is <4 is very important for maintaining human health and preventing and managing obesity. Therefore, consumers should focus on the intake of OR and PFR in rice and other foods.

#### 3.2.2. Comparison of VitE Content in Rice Grains According to Rice Types with the Same DOM

**Figure 6:**
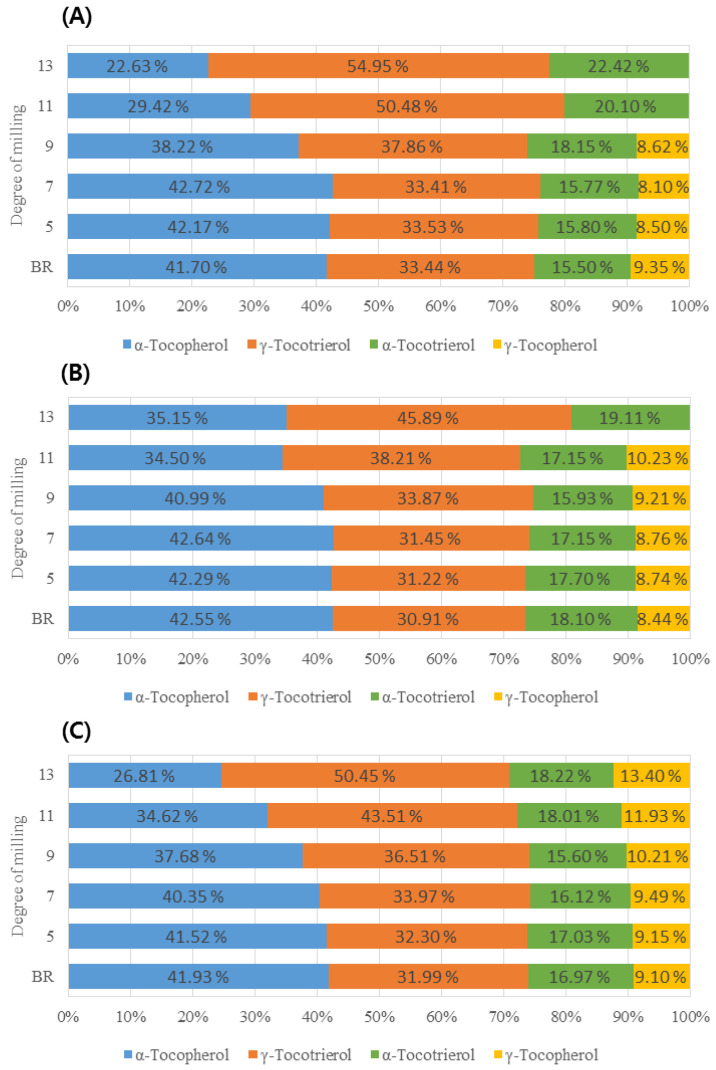
VitE composition and proportion of (**A**) OR, (**B**) PFR, and (**C**) CR according to the DOM. The percentage of each vitamin E indicates the ratio of the content of each vitamin E to the total vitamin E content.

The content and proportion of VitE according to rice types with the same DOM are shown in Table S7. The total VitE content significantly differed in the OR, PFR, and CR samples with the same DOM, and the PFR samples exhibited larger amounts of VitE compared to OR or CR samples (Table S7). The main VitEs such as αT and γT_3_ contributed to >70% of the total VitE content in all rice types at all DOM levels. The αT and total VitE levels in all rice types decreased significantly by around 63% and 53% in 11 DOM rice as compared to the level in BR (*p* < 0.05). In particular, the αT and γT_3_ levels in OR were further decreased by more than 10% in 11 DOM compared to that in the PFR and CR samples (Table S7). Additionally, γT was not found in the OR and PFR samples with a DOM >11 (Figure 6). Similarly, the proportion of VitE, particularly αT and γT, appeared to change by <20% in all rice types with increasing DOM levels, and large changes were observed in OR and CR compared to in PFR (Figure 6).

Notably, rice bran has an abundant VitE content of up to 300 mg·kg^−1^, and αT, γT_3_, γT, and αT_3_ appear to be major forms [26]. According to a previous study [3], the mean VitE content in non-pigmented whole rice grain (60.2 µg·g^−1^) was higher than that in pigmented whole grain (53.1 µg·g^−1^). Additionally, the VitE homolog composition and content of rice grain widely varied in rice cultivars and rice fractions. γT_3_ constituted the largest proportion of the total VitE content (27–63%), followed by αT (10–30%), αT_3_ (9–19%), γT (9–14%), δT_3_ (2–6%), βT_3_ (1–4%), βT (1–2%), and δT (1–2%). According to previous studies [17,27,28], the αT content in BR varied in Basmati BR (12.1 µg·g^−1^), Jaya BR (9.9 µg·g^−1^), and Venezulelan rice (< 8.5 µg·g^−1^); these differences may be associated with the effects of the cultivar and environmental/cultivation conditions. The αT content (<8.0 µg·g^−1^ in BR) of OR, PFR, and CR in this study were similar to those observed in previous studies. Additionally, BR typically has a 2-fold higher αT content as observed in milled rice, and our findings showed that the αT content was decreased by around 50% in all rice types with a DOM >11.

To our knowledge, few comparative studies of VitE profiling in rice grains grown using different cultivation methods (i.e., organic, green, or conventional method) have been performed. In a prior study [29], as compared to levels in conventional barley, the levels of tocotrienol homologs in organic Greek barley cultivars were significantly increased by <37.14% for αT_3_, <41.09% for (β + γ)T_3_, and <196.61% for δT_3_, whereas the levels of four tocopherol homologs were decreased. However, in this study, larger amounts of the four VitE homologs were observed in PFR and CR compared to in OR at all DOM levels. These differences may be attributed to the complicated associations of species, cultivars, cultivation methods, environments, and combinations. Thus, a more comprehensive study is needed to describe the impact of cultivation methods (organic vs. conventional) on phytochemicals such as VitE in rice grain fractions such as bran, pericarp, and aleurone.

#### 3.2.3. Phenolic Level Comparison According to the Rice Types with the Same DOM

The effects of rice types according to the DOM on the differences in phenolic content are summarized in Table S8. The major phenolics (i.e., ferulic, *p*-coumaric, and *p*-hydroxybenzoic acid) and sum of phenolics found in rice grains did not significantly differ with the rice type at the same DOM levels. Interestingly, the levels of major phenolics were affected by the DOM of rice grains but the effect of the rice type was significantly low, whereas the levels of minor phenolics (i.e., protocatechuic, gentisic, caffeic acid) were shown to be affected by the rice type rather than by the DOM (Table S8).

Phenolic compounds of plants are well-known as protective components produced in response to external stress. The differences between organic and conventional farming methods with regard to nutritional/bioactive values in agroproducts remain unclear because of several factors such as the region, variety, cultivation, and soil condition [30]. However, the C/N balance and growth/differentiation balance theories enable description of the effect of cultivation methods on the differences in nutritional and bioactive compounds. Thus, the low nitrogen availability commonly observed with organic farming methods is the most common growth-limiting condition in natural ecosystems; these levels can be increased to increase the production of carbon-containing defensive compounds such as phenolics and terpenoids [31].

However, the phenolic levels in this study were not significantly different in rice grains with the same DOM. When they were disregarded as an effect caused by the DOM, the OR samples had a slightly higher total phenolic content compared to that in CR. Our results were partly consistent with those of prior studies revealing limited differences in phenolics found in wheat, oats, and rice produced organically and conventionally [30,32]. Notably, ferulic and *p*-coumaric acids were the major phenolics observed in rice grains; these compounds typically exist in free, soluble conjugated, or insoluble bound forms. Particularly, the bound form involves formation of a cell wall structure [18]. According to previous studies, the levels of ferulic acid found in BR were approximately 4-fold higher than those in milled (or white) rice [21,33]. Because ferulic acid was present in the bound form in rice grains, its extraction efficiency was important for accurately determining the ferulic acid content in rice. Here, ferulic acid was also observed as a main phenolic compound in rice grains at all DOM levels, accounting for 42–46% to the total measured phenolics. Unlike the previous reports described above, however, its content differed by only 19% in rice grains of BR and 13 DOM rice. The ferulic acid level in CR was decreased by 22% between BR and 13 DOM and appeared to decrease to 15% in OR (*p* < 0.05, Table S8). However, this difference may not be easy to explain because the ferulic content in rice grains can be affected by several factors, such as cultivars, cultivation methods, geo-climatic features, and soil.

### 3.3. Comparison of DPPH Free Radical Scavenging Activity According to Rice Type and DOM

Several assays such as radical scavenging activity (i.e., hydroxyl radical, superoxide anion, nitric oxide), ferric reducing antioxidant power assay, β-carotene bleaching assay, and phosphomolybdenum assay for total antioxidant capacity have been reported to evaluate antioxidative activity in various agricultural products [34,35,36]. Among these methods, the DPPH method has been widely used for antioxidative activity evaluation because of its easy, simple, and economic evaluation process as well as broad applicability to hydrophilic and lipophilic molecules. DPPH reacts with the entire sample, even with weak antioxidants, after sufficient time [37]. However, the DPPH method has some limitations such as the steric hindrance of larger molecules with radical location in the DPPH chemical structure [38]. Therefore, in this study, the antioxidative activity of rice depending on DOM was preliminarily tested using the DPPH method.

**Figure 7:**
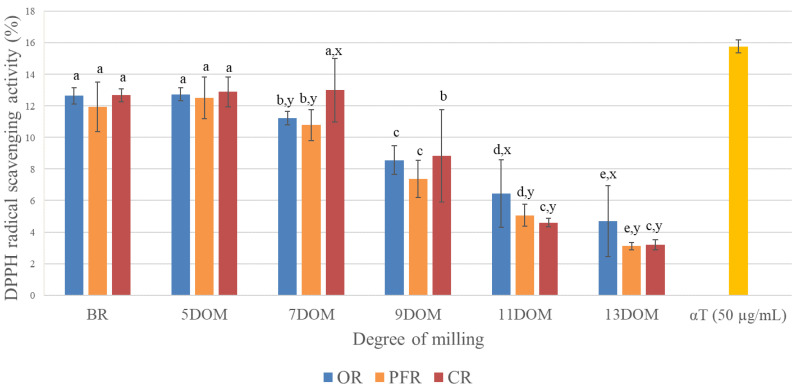
DPPH-free radical scavenging activity by rice type depending on the degree of milling. Different superscripts represent a significant difference depending on the DOM (a–e, *p* < 0.05) and rice type (x and y, *p* < 0.05).

The DPPH free radical scavenging activity of the rice extract decreased with the further milling of rice grains; DPPH activity significantly decreased in (by about 60%) rice with a DOM between 7 and 11 as compared to that in BR (Figure 2D). Additionally, three rice types showed significantly decreasing trends in DPPH activity with increased DOM levels; in the BR to 9 DOM samples, the rice type did not affect DPPH scavenging activity (Figure 7). However, for 11 and 13 DOM rice, OR showed a stronger DPPH scavenging activity than did PFR and CR (*p* < 0.05, Figure 7). The αT standard used as a positive control appeared to have DPPH radical scavenging activity of 15.8% (50 µg/mL), 32.3% (100 µg/mL), and 67.2% (200 µg/mL), respectively (Figure S4). With regard to the rice type and DOM, all rice sample extracts showed lower DPPH activity than the positive control (αT) of 50 µg/mL (Figure 2D and Figure 7).

**Figure 8:**
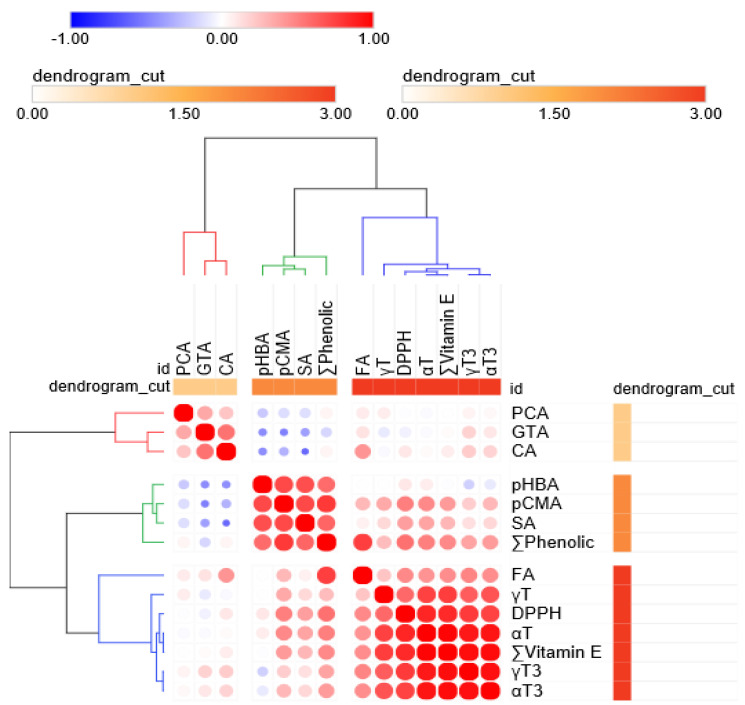
Hierarchical cluster correlation matrix of vitamin E and phenolic compounds in ecofriendly and conventional rice samples. Each circle indicates the Pearson’s correlation coefficient for a pair of compounds, and the correlation coefficient values are represented by the intensity of the blue (*r* = −1) or red color (*r* = 1), in combination with the circle size, as indicated by the color and size scale.

In addition, a hierarchical cluster Pearson’s correlation analysis was conducted for the VitE, phenolic, and DPPH levels measured in all OR, PFR, and CR samples. Three clusters were formed in hierarchical cluster analysis; one group mostly showed the VitE homologs and DPPH activity and the other two clusters showed all other phenolics found in rice grain, except for ferulic acid (Figure 8). Further, αT (*r* = 0.854, *p* < 0.0001) showed the strongest correlation with DPPH scavenging activity, followed by total VitE (*r* = 0.840), γT_3_ (*r* = 0.773), αT_3_ (*r* = 0.743), and γT (*r* = 0.591). Additionally, total phenolic (*r* = 0.557) and *p*-coumaric acid (*r* = 0.501) levels were significantly correlated with DPPH activity. Other compounds found in rice samples appeared to show weak or no correlation with DPPH activity in this study (Figure 8). *p*-Coumaric and ferulic acids showed a strong correlation with the total phenolic content in rice samples. The αT, αT_3_, and γT_3_ levels were very highly correlated with the total VitE level (*r* > 0.95). Interestingly, the αT content only showed a correlation with the total phenolic content among the VitE and phenolic groups found in rice grain depending on the DOM.

VitE homologs and phenolics present in various crops are known to have certain antioxidant activities such as the free-radical scavenging ability, and have a positive effect on human health by reducing the risk of disease [30,39,40]. For example, a free hydroxyl group on the chromanol ring of the VitE structure is responsible for its antioxidant properties, and hydrogen atoms from this group can be donated to free radicals, resulting in a resonance-stabilized VitE radical [26].

Notably, ferulic or *p*-coumaric acid was the most abundant phenolic in the bran layer or pericarp of rice grains [17,20]. Moreover, the amount of αT was 4-fold higher in the rice bran layer than in the whole grain [3]. Therefore, the antioxidant activity was highly affected by the composition/content of nutritional and bioactive compounds present in rice grains, particularly the bran layer, depending on the DOM. In a prior study [20], the pericarp and embryo fractions of rice grains contributed to approximately 30% of the total antioxidant activity, despite their small weight in proportion (4.6%) to whole BR. Moreover, the BR extract showed approximately 2-fold higher DPPH radical scavenging activity than milled rice or its fractions [17].

Our results also showed that the BR had >2-fold higher free-radical scavenging activity than that of polished rice with a DOM > 11. Identification of αT, ferulic, and *p*-coumaric acids revealed the major phytochemicals in rice grains, despite their varying contents based on their DOM levels. A previous study [3] showed that phenolic acids had up to 4-fold higher antioxidant activities than αT and γ-oryzanol. However, in this study, the αT (*r* = 0.854) level exhibited a stronger correlation with DPPH activity than phenolics (*r* ≤ 0.501). The sample matrix used in this study may have included antioxidant components that reacted slowly or were even inert to the DPPH radical. Hence, a more accurate evaluation of the antioxidant activity according to the rice type and DOM may require additional sample analysis and different antioxidant assays.

### 3.4. Discriminant Analysis According to Rice Type and DOM

DA is a supervised pattern recognition method that maximizes the ratio of inter-class variance and minimizes that of intra-class variance; hence, it minimizes the possibility of misclassifying cases. Therefore, many previous studies have reported the suitability of the DA approach for determining the geographical origin and organic of various foodstuffs [41,42,43].

**Figure 9:**
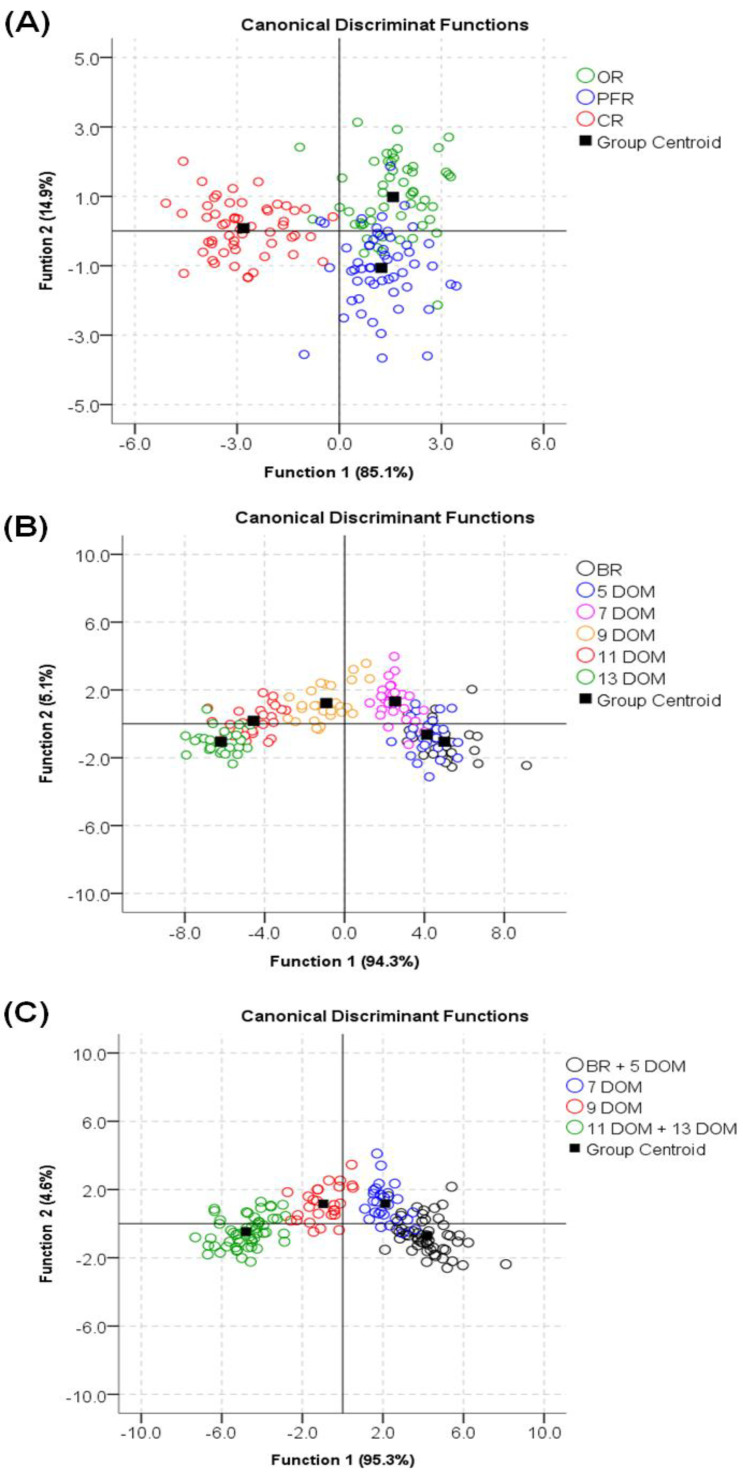
Stepwise discriminant analysis (SDA) using the FA, vitE, and phenolic metabolite contents measured in rice grain. (**A**) rice type identification, (**B**) six levels of DOM identification, (**C**) four levels of DOM identification.

In this study, SDA was conducted to classify and predict the rice type (i.e., OR, PFR, CR) using 12 variables among a total of 21 variables (FA, VitE, and phenolics). During rice type identification (Figure 9A), the log determinants were slightly different, and Box’s M (1009.091, F = 5.780 *p* < 0.000) indicated that the assumption of equality of covariance matrices had been violated. However, for large samples, this problem is typically not considered to be serious. The first two canonical correlations of 0.896 and 0.645 indicate that the model explained 80.3% and 41.6% of the variations observed in the grouping variables, respectively. The Wilks’ lambda value (0.116) indicated that the two discriminant functions were highly significant (*p* < 0.0000), meaning that 11.6% remained unexplained (see Supplementary SDA Tables). The major contributors to discriminating the three rice types were C18:1n-9 (standardized coefficient, −4.261), αT (1.889), and C20:0 (1.635) in the first function, and αT (−2.426), C18:1n-9 (2.297), and C18:3n3 (1.891) in the second function (see Supplementary SDA Tables). Thus, the results of original and cross-validated classification, respectively, showed that, overall, 87.0% and 81.5% rice types were correctly classified (Table 1, Figure 9A).

We also developed and tested a discriminant model for six levels of rice DOM identification (BR, 5, 7, 9, 11, and 13 DOM) using the SDA method and eight of 21 variables (Table S9, Figure 9B). However, the accuracy of classification for the original set (79.0%) and cross-validated set (74.1%) was low. Therefore, we developed another SDA model for rice DOM identification using the four levels (i.e., BR + 5 DOM, 7 DOM, 9 DOM, and 11 + 13 DOM) with 7 of the 21 variables (Figure 9C). The first two functions were described by 99.8% of the total variance, showing canonical correlations of 0.968 and 0.645, respectively. Three discriminant functions were highly significant (*p* < 0.0000), with the Wilks’ lambda value (0.036) showing that 3.6% remained unexplained (see Supplementary SDA Tables). The major contributors to rice DOM identification were αT (1.181) in the first function, C24:0 (−1.733) in the second function, and protocatechuic acid (0.883) in the third function (see Supplementary SDA Tables). Consequently, the results of original and cross-validated classification showed that, overall, 93.8% and 92.6% were correctly classified (Table 2, Figure 9C).

In general, rice grains with a DOM of more than 10 are considered as white (polished) rice. Our current findings were similar, showing that for polished rice, it was rather difficult to discriminate between the 11 and 13 DOM rice samples (Figure 9B); however, if they were considered to belong to the same polished rice group (11 + 13 DOM), rice DOM identification was more accurate against other groups (Table 2, Figure 9C). This was also observed in the case of BR and 5 DOM identification. Consequently, nutraceutical analysis of rice grains combined with SDA enabled us to effectively describe the nutritional features and differences in rice grains according to the DOM. Furthermore, our results revealed the applicability of rice type identification for determining the organic authenticity of rice.

## 4. Conclusions

In summary, the DOM of rice grains significantly affected their nutraceutical content and antioxidant activity as compared to the rice type or a combination of the DOM and rice type. In particular, the antioxidant activity and nutritionally desirable UFA (i.e., oleic and linoleic acids) and αT levels were significantly decreased in polished rice varieties with a DOM between 7 and 9. Because we used a mixed rice sample obtained from common retail markets, in which rice cultivars were not specified, there was no a clear trend in the variations in nutraceuticals and antioxidant activities among OR, PFR, and CR; however, polished rice (>11 DOM) and OR samples exhibited higher antioxidant activity than that of PFR and CR samples. αT (*r* = 0.854, *p* < 0.0001), γT_3_ (*r* = 0.773), and *p*-coumaric acid (*r* = 0.501), the major nutraceuticals in rice, were highly correlated with DPPH free radical scavenging activity. The SDA model was used to accurately perform original and cross-validated classification of 87.0% and 81.5% of rice types, respectively; hence, it is feasible to use this approach to identify the organic authenticity of rice. Furthermore, SDA showed four levels of similarity/dissimilarity during rice DOM classification, and its accuracy for cross-validated samples was 92.6%. Overall, our findings further contribute to the understanding of the differences and variations in the nutraceutical content and antioxidant activity in different types of rice grain and DOM and provide important insights into the nutritional evaluation of rice grains, which is a staple crop for nearly half of the world’s population.

## Figures and Tables

**Table 1:** Classification and cross-validated results of rice types (OR, PFR, and CR) identified using SDA.

		Rice Type	Predicted Group Membership			Total
			OR	PFR	CR	
Original ^a^	Count	OR	43	9	2	54
		PFR	8	46	0	54
		CR	1	1	52	54
	%	OR	79.6	16.7	3.7	100.0
		PFR	14.8	85.2	0	100.0
		CR	1.9	1.9	96.3	100.0
Cross-validated ^b,c^	Count	OR	39	13	2	54
		PFR	11	41	2	54
		CR	1	1	52	54
	%	OR	72.2	24.1	3.7	100.0
		PFR	20.4	75.9	3.7	100.0
		CR	1.9	1.9	96.3	100.0

^a^ 87.0% of original grouped cases were correctly classified. ^b^ Cross validation is done only for those cases in the analysis. In cross validation, each case is classified by the functions derived from all cases other than that case. ^c^ 81.5% of cross-validated grouped cases were correctly classified.

**Table 2:** Classification and cross-validation results of rice DOMs (4 levels) identified using SDA.

		DOM Level	Predicted Group Membership				Total
			BR + 5 DOM	7 DOM	9 DOM	11 DOM + 13 DOM	
Original ^a^	Count	BR + 5 DOM	50	4	0	0	54
		7 DOM	4	23	0	0	27
		9 DOM	0	1	26	0	27
		11 DOM + 13 DOM	0	0	1	53	54
	%	BR + 5 DOM	92.6	7.4	0	0	100.0
		7 DOM	14.8	85.2	0	0	100.0
		9 DOM	0	3.7	96.3	0	100.0
		11 DOM + 13 DOM	0	0	1.9	98.1	100.0
Cross-Validated ^b^^,c^	Count	BR + 5 DOM	50	4	0	0	54
		7 DOM	5	22	0	0	27
		9 DOM	0	2	25	0	27
		11 DOM + 13 DOM	0	0	1	53	54
	%	BR + 5 DOM	92.6	7.4	0	0	100.0
		7 DOM	18.5	81.5	0	0	100.0
		9 DOM	0	7.4	92.6	0	100.0
		11 DOM + 13 DOM	0	0	1.9	98.1	100.0

^a^ 93.8% of original grouped cases were correctly classified. ^b^ Cross validation is done only for those cases in the analysis. In cross validation, each case is classified by the functions derived from all cases other than that case. ^c^ 92.6% of cross-validated grouped cases were correctly classified.

## Supplementary Materials

The following are available online at https://www.mdpi.com/2304-8158/9/9/1297/s1, Figure S1: Representative gas chromatography-flame ionization detector (GC-FID) chromatograms: (**A**) 37 FAME standards mixture, (**B**) OR (a brown rice type), (**C**) The same OR chromatogram spiked with fatty acid standards (C14:0, C17:0, C20:0, C20:1, C24:0) 1; C4:0, 2; C6:0, 3; C8:0, 4; C10:0, 5; C11:0, 6; C12:0, 7; C13:0, 8; C14:0, 9; C14:1, 10; C15:0, 11; C15:1, 12;C16:0, 13; C16:1, 14; C17:0, 15; C17:1, 16; C18:0, 17 + 18; C18:1n9 t and c, 19, 20; C18:2n6 t and c, 21; C18:3n6, 22; C18:3n3, 23; C20:0, 24; C20:1n9, 25; C20:2, 26 + 30; C20:3n6 and C21:0, 27; C20:3n3, 28; C20:4n6, 29; C20:5n3, 31; C22:0, 32; C22:1n9, 33; C22:2, 35: C23:0, 36; C24:0, 34 + 37; C22:6n3 and C24:1n9, Figure S2: Representative GC-FID chromatogram of the mixture of eight tocopherol and tocotrienol standards (red solid line) and OR with brown rice type (black solid line). α, β, γ, δT; α-, β-, γ-, δ-tocopherol, α, β, γ, δT3; α-, β-, γ-, δ-tocotrienol, Figure S3: Representative multiple reaction monitoring (MRM) ion chromatogram of standards of 54 selected phenolic compounds; (**A**), total and extracted ion chromatograms of brown rice and organic rice (**B**), and MS/MS spectra and fragmentation of the representative phenolic standards (**C**) 1. Gallic acid; 2. 5-Sulfosalicylic acid; 3. Homogentisic acid; 4. Peonidin 3-*O*-glucoside chloride; 5. Protocatechuic acid; 6. Delphinidin chloride; 7. Chlorogenic acid; 8. Catechin; 9. Cyanidin chloride; 10. Daidzin; 11. Glycitin; 12. Orientin; 13. Pelargonidin chloride; 14. Malvidin chloride; 15. Peonidin chloride; 16. Rutin; 17. *p*-Hydroxybenzoic acid; 18. Caffeic acid; 19. Syringic acid; 20. Vitexin; 21. Gentisic acid; 22. Polydatin; 23. Malonyl glycitin; 24. Malonyl daidzin; 25. Genistin; 26. Naringin; 27. β-Resorcylic acid; 28. Acetyl daidzin; 29. Acetyl glycitin; 30. *p*-Coumaric acid; 31. Vanillic acid; 32. Ferulic acid; 33. Malonyl genistin; 34. Vanillin; 35. Veratric acid; 36. *m*-Coumaric acid; 37. Myricetin; 38. Acetyl genistin; 39. *o*-Coumaric acid; 40. *trans*-Resveratrol; 41. Daidzein; 42. Glycitein; 43. Luteolin; 44. Quercetin; 45. Salicylic acid; 46. *cis*-Resveratrol; 47. *trans*-Cinnamic acid; 48. Apigenin; 49. Naringenin; 50. Genistein; 51. Kaempferol; 52. Hesperetin; 53. Formononetin; 54. Biochanin A. Figure S4: DPPH free radical scavenging activity according to three concentration levels of α-tocopherol. Table S1: Optimized multiple reaction monitoring parameters of 54 selected phenolic compounds, Table S2: Calibration curves of nine phenolic compounds detected in the examined rice samples, Table S3: Dependence of fatty acid composition and content in rice samples on the degree of milling (DOM) and rice type (mg·g^−1^ on a dry weight basis), Table S4: Dependence of vitamin E composition and content in rice samples on the degree of milling (DOM) and rice type (µg·g^−1^ on dry weight basis), Table S5: Dependence of phenolic composition and content of rice samples on the degree of milling (DOM) and rice type (µg·g^−1^ on dry weight basis), Table S6: Fatty acid composition and content (mg·g^−1^ on dry weight basis) in rice types with the same degree of milling (DOM), Table S7: Vitamin E composition and content (µg·g^−1^ on dry weight basis) in rice types with the same degree of milling (DOM), Table S8: Phenolic composition and content (µg·g^−1^ on dry weight basis) in rice types with the same degree of milling (DOM), Table S9: Classification and cross-validated results ^a,b^ for rice DOM identification (six levels) using SDA.
