# Supplementary material for: The Effect of Degree of Milling on the Nutraceutical Content in Ecofriendly and Conventional Rice (Oryza sativa L.)
Source: Foods. 2020 Sep 15;9(9):1297. doi: 10.3390/foods9091297 (PMC7555660; doi:10.3390/foods9091297)
Supplement: Supplementary file 1 [file foods-09-01297-s001.zip › foods-906860-Supp.docx]

**Electronic Supplementary Information (ESI) for Foods**

The Effect of Degree of Milling on the Nutraceutical Content in Ecofriendly and Conventional Rice (*Oryza sativa* L.)

Seung-Hyun Kim^1^, Yu-jin Yang^1^, and Ill-Min Chung^*^

Department of Crop Science, College of Sanghuh Life Science, Konkuk University, 120 Neungdong-ro, Gwangjin-gu, Seoul 05029, Republic of Korea; kshkim@konkuk.ac.kr (S-H.K.); jin9031@konkuk.ac.kr (Y.-J.Y.); imcim@konkuk.ac.kr (I.-M.C.)

^1^ These authors contributed equally to this study.

***** Correspondence: [imcim@konkuk.ac.kr](mailto:imcim@konkuk.ac.kr); Tel.: +82-2-450-3730; Fax: 82-2-446-7856

Received: date; Accepted: date; Published: date

Table S1. Optimized multiple reaction monitoring parameters of 54 selected phenolic compounds

| **Class** | **Subclass** | **Compound** | **Formula** | **Q1 [M-H]^-^** | **Q3** | **DP** | **FP** | **EP** | **CEP** | **CE** | **CXP** |
| --- | --- | --- | --- | --- | --- | --- | --- | --- | --- | --- | --- |
|  |  |  |  | **(*m/z*, Da)** | **(*m/z*, Da)** | **(volt)** | **(volt)** | **(volt)** | **(volt)** | **(volt)** | **(volt)** |
| Phenolic acid | Hydroxybenzoic acid | Salicylic acid | C_7_H_6_O_3_ | 136.9 | 92.9 | -16.0 | -320.0 | -7.5 | -12.0 | -22.0 | -6.0 |
|  |  | *p*-Hydroxybenzoic acid | C_7_H_6_O_3_ | 136.9 | 92.9 | -16.0 | -350.0 | -8.5 | -12.0 | -24.0 | -6.0 |
|  |  | Gentisic acid | C_7_H_6_O_4_ | 152.9 | 107.9 | -16.0 | -350.0 | -8.5 | -6.0 | -28.0 | -22.0 |
|  |  | Protocatechuic acid | C_7_H_6_O_4_ | 152.9 | 108.9 | -16.0 | -330.0 | -9.0 | -10.0 | -22.0 | -6.0 |
|  |  | *β*-Resorcylic acid | C_7_H_6_O_4_ | 152.9 | 108.9 | -21.0 | -350.0 | -4.5 | -10.0 | -18.0 | -6.0 |
|  |  | Gallic acid | C_7_H_6_O_5_ | 169.1 | 125.0 | -26.0 | -350.0 | -10.0 | -8.0 | -20.0 | -6.0 |
|  |  | 5-Sulfosalicylic acid | C_7_H_6_O_6_S | 216.8 | 198.8 | -21.0 | -320.0 | -4.5 | -10.0 | -22.0 | -8.0 |
|  |  | Vanillic acid | C_8_H_8_O_4_ | 166.9 | 151.9 | -11.0 | -250.0 | -6.5 | -10.0 | -18.0 | -32.0 |
|  |  | Veratric acid | C_9_H_10_O_4_ | 181.0 | 136.9 | -16.0 | -350.0 | -7.5 | -12.0 | -18.0 | -8.0 |
|  |  | Syringic acid | C_9_H_10_O_5_ | 196.9 | 182.0 | -16.0 | -350.0 | -10.5 | -12.0 | -30.0 | -6.0 |
|  | Hydroxycinnamic acid | *trans*-Cinnamic acid | C_9_H_8_O_2_ | 146.9 | 102.9 | -26.0 | -310.0 | -7.0 | -10.0 | -14.0 | -20.0 |
|  |  | *o*-Coumaric acid | C_9_H_8_O_3_ | 162.8 | 118.9 | -6.0 | -290.0 | -11.5 | -14.0 | -18.0 | -4.0 |
|  |  | *m*-Coumaric acid | C_9_H_8_O_3_ | 162.8 | 119.0 | -21.0 | -330.0 | -9.0 | -8.0 | -22.0 | -4.0 |
|  |  | *p*-Coumaric acid | C_9_H_8_O_3_ | 162.8 | 118.7 | -11.0 | -340.0 | -7.0 | -14.0 | -20.0 | -6.0 |
|  |  | Caffeic acid | C_9_H_8_O_4_ | 178.9 | 134.8 | -16.0 | -330.0 | -8.0 | -14.0 | -22.0 | -6.0 |
|  |  | Ferulic acid | C_10_H_10_O_4_ | 192.9 | 133.9 | -11.0 | -350.0 | -10.0 | -12.0 | -22.0 | -8.0 |
|  |  | Chlorogenic acid | C_16_H_18_O_9_ | 352.8 | 191.0 | -21.0 | -330.0 | -7.0 | -32.0 | -30.0 | -10.0 |
|  | Phenylacetic acid | Homogentisic acid | C_8_H_8_O_4_ | 166.9 | 122.9 | -21.0 | -300.0 | -7.0 | -14.0 | -14.0 | -28.0 |
| Flavonoid | Flavone | Apigenin | C_15_H_10_O_5_ | 268.9 | 117.0 | -46.0 | -350.0 | -10.0 | -20.0 | -56.0 | -8.0 |
|  |  | Luteolin | C_15_H_10_O_6_ | 285.0 | 133.2 | -66.0 | -250.0 | -10.0 | -16.0 | -46.0 | -30.0 |
|  |  | Vitexin | C_21_H_20_O_10_ | 430.8 | 310.8 | -56.0 | -260.0 | -9.0 | -28.0 | -22.0 | -50.0 |
|  |  | Orientin | C_21_H_20_O_11_ | 447.1 | 327.0 | -61.0 | -220.0 | -11.0 | -20.0 | -22.0 | -54.0 |
|  | Flavonol | Kaempferol | C_15_H_10_O_6_ | 284.8 | 65.0 | -81.0 | -270.0 | -8.5 | -20.0 | -72.0 | -12.0 |
|  |  | Quercetin | C_15_H_10_O_7_ | 300.9 | 150.8 | -31.0 | -330.0 | -10.5 | -18.0 | -28.0 | -32.0 |
|  |  | Myricetin | C_15_H_10_O_8_ | 316.8 | 151.1 | -61.0 | -310.0 | -10.0 | -16.0 | -36.0 | -28.0 |
|  |  | Rutin | C_27_H_30_O_16_ | 609.0 | 299.7 | -91.0 | -350.0 | -10.5 | -34.0 | -52.0 | -14.0 |
|  | Flavanone | Naringenin | C_15_H_12_O_5_ | 270.8 | 64.7 | -66.0 | -90.0 | -8.0 | -16.0 | -30.0 | -26.0 |
|  |  | Hesperetin | C_16_H_14_O_6_ | 301.0 | 163.7 | -51.0 | -310.0 | -7.5 | -20.0 | -32.0 | -32.0 |
|  |  | Naringin | C_27_H_32_O_14_ | 579.0 | 151.1 | -126.0 | -310.0 | -10.5 | -28.0 | -52.0 | -32.0 |
|  | Flavanol | Catechin | C_15_H_14_O_6_ | 288.8 | 108.9 | -21.0 | -340.0 | -9.0 | -16.0 | -34.0 | -4.0 |

| **Class** | **Subclass** | **Compound** | **Formula** | **Q1 [M-H]^-^** | **Q3** | **DP** | **FP** | **EP** | **CEP** | **CE** | **CXP** |
| --- | --- | --- | --- | --- | --- | --- | --- | --- | --- | --- | --- |
|  |  |  |  | **(*m/z*, Da)** | **(*m/z*, Da)** | **(volt)** | **(volt)** | **(volt)** | **(volt)** | **(volt)** | **(volt)** |
| Flavonoid | Anthocyanin | Pelargonidin | C_15_H_11_ClO_5_ | 305.0 | 268.9 | -1.0 | -270.0 | -4.0 | -14.0 | -12.0 | -52.0 |
|  |  | Cyanidin | C_15_H_11_ClO_6_ | 320.9 | 284.9 | -1.0 | -290.0 | -4.5 | -16.0 | -10.0 | -54.0 |
|  |  | Delphinidin | C_15_H_11_ClO_7_ | 336.9 | 300.8 | -1.0 | -290.0 | -4.5 | -14.0 | -10.0 | -56.0 |
|  |  | Peonidin | C_16_H_13_ClO_6_ | 334.9 | 298.8 | -1.0 | -330.0 | -3.0 | -14.0 | -12.0 | -56.0 |
|  |  | Malvidin | C_17_H_15_ClO_7_ | 365.0 | 328.9 | -6.0 | -350.0 | -3.5 | -14.0 | -14.0 | -10.0 |
|  |  | Peonidin 3-*O*-glucoside | C_22_H_23_ClO_11_ | 497.0 | 298.9 | -1.0 | -350.0 | -8.0 | -26.0 | -32.0 | -54.0 |
|  | Isoflavone | Daidzein | C_15_H_10_O_4_ | 252.9 | 131.9 | -76.0 | -350.0 | -10.5 | -26.0 | -54.0 | -6.0 |
|  |  | Genistein | C_15_H_10_O_5_ | 268.9 | 132.9 | -76.0 | -240.0 | -10.5 | -12.0 | -44.0 | -6.0 |
|  |  | Glycitein | C_16_H_12_O_5_ | 282.9 | 267.8 | -31.0 | -340.0 | -7.0 | -54.0 | -26.0 | -12.0 |
|  |  | Formononetin | C_16_H_12_O_4_ | 266.8 | 251.8 | -21.0 | -290.0 | -7.5 | -14.0 | -24.0 | -44.0 |
|  |  | Biochanin A | C_16_H_12_O_5_ | 282.8 | 267.9 | -46.0 | -210.0 | -8.0 | -14.0 | -24.0 | -48.0 |
|  |  | Daidzin | C_21_H_20_O_9_ | 415.0 | 251.9 | -66.0 | -320.0 | -10.0 | -28.0 | -38.0 | -46.0 |
|  |  | Genistin | C_21_H_20_O_10_ | 431.1 | 268.0 | -81.0 | -140.0 | -9.5 | -64.0 | -44.0 | -12.0 |
|  |  | Glycitin | C_22_H_22_O_10_ | 445.1 | 238.7 | -81.0 | -300.0 | -8.0 | -22.0 | -42.0 | -52.0 |
|  |  | Acetyl Daidzin | C_23_H_22_O_10_ | 456.9 | 252.0 | -86.0 | -350.0 | -10.0 | -54.0 | -44.0 | -12.0 |
|  |  | Acetyl Genistin | C_23_H_22_O_11_ | 473.0 | 267.9 | -101.0 | -230.0 | -10.5 | -30.0 | -40.0 | -8.0 |
|  |  | Acetyl Glycitin | C_24_H_24_O_11_ | 487.0 | 281.9 | -76.0 | -350.0 | -10.5 | -30.0 | -36.0 | -10.0 |
|  |  | Malonyl Daidzin | C_24_H_22_O_12_ | 501.0 | 252.7 | -6.0 | -330.0 | -4.0 | -21.0 | -22.0 | -50.0 |
|  |  | Malonyl Genistin | C_24_H_22_O_13_ | 517.0 | 268.7 | -11.0 | -340.0 | -4.5 | -22.0 | -20.0 | -8.0 |
|  |  | Malonyl Glycitin | C_25_H_24_O_13_ | 531.0 | 282.9 | -26.0 | -340.0 | -5.5 | -21.8 | -22.0 | -48.0 |
| Stilbenoid | Stilbene | *cis*-Resveratrol | C_14_H_12_O_3_ | 226.9 | 142.8 | -61.0 | -330.0 | -10.5 | -14.2 | -34.0 | -28.0 |
|  |  | *trans*-Resveratrol | C_14_H_12_O_3_ | 226.9 | 142.8 | -56.0 | -170.0 | -11.0 | -14.0 | -34.0 | -32.0 |
|  |  | Polydatin | C_20_H_22_O_8_ | 389.0 | 226.8 | -71.0 | -320.0 | -10.5 | -18.2 | -20.0 | -48.0 |
| Phenolic aldehyde | Benzaldehyde | Vanillin | C_8_H_8_O_3_ | 150.9 | 135.9 | -16.0 | -280.0 | -4.0 | -10.0 | -18.0 | -28.0 |

Q1: Precursor ion mass, Q3: Product ion mass, DP: Declustering potential, FP: Focusing potential, EP: Entrance potential, CEP: Collision cell entrance potential, CE: Collision energy, CXP: Collision cell exit potential

Table S2. Calibration curves of nine phenolic compounds detected in the examined rice samples

| Class | Subclass | Compound | RT | Regression Equation | R2 | Linearity Range (µg/mL) | LOD (µg/mL) | LOQ (µg/mL) |
| --- | --- | --- | --- | --- | --- | --- | --- | --- |
| Phenolic acid | Hydroxybenzoic acid | Salicylic acid | 16.19 | y = 8E+06x + 34320 | 1 | 0.01-0.5 | 0.001 | 0.005 |
|  |  | *p*-Hydroxybenzoic acid | 9.81 | y = 989604x + 3480.5 | 1 | 0.005-0.5 | 0.009 | 0.029 |
|  |  | Gentisic acid | 10.36 | y = 1E+06x – 22964 | 1 | 0.01-0.5 | 0.007 | 0.022 |
|  |  | Protocatechuic acid | 7.86 | y = 2E+06x – 92528 | 1 | 0.005-5 | 0.006 | 0.019 |
|  |  | Veratric acid | 12.83 | y = 5638.1x + 4264 | 0.99 | 1-20 | 1.034 | 3.448 |
|  | Hydroxycinnamic acid | *p*-Coumaric acid | 11.87 | y = 3E+06x + 55558 | 0.99 | 0.005-0.5 | 0.003 | 0.01 |
|  |  | Caffeic acid | 9.9 | y = 4E+06x + 9565.4 | 1 | 0.005-0.1 | 0.003 | 0.009 |
|  |  | Ferulic acid | 12.31 | y = 116437x + 2071.7 | 1 | 0.005-1 | 0.037 | 0.123 |
| Phenolic aldehyde | Benzaldehyde | Vanillin | 12.54 | y = 90094x - 340.35 | 1 | 0.1-1 | 0.071 | 0.236 |

RT: Retention time, R2: Coefficient of determination, LOD: Limit of detection, LOQ: Limit of quantitation

Table S3. Dependence of fatty acid composition and content in rice samples on the degree of milling (DOM) and rice type (mg·g^-1^ on dry weight basis).

| Fatty acid | Degree of milling (D) | | | | | |  | Rice type (T) | | |  | *p*-Value | | |
| --- | --- | --- | --- | --- | --- | --- | --- | --- | --- | --- | --- | --- | --- | --- |
|  | Brown rice | 5 DOM | 7 DOM | 9 DOM | 11 DOM | 13 DOM |  | OR | PFR | CR |  | Main factor | | Interaction |
|  | n=27 | n=27 | n=27 | n=27 | n=27 | n=27 |  | n=54 | n=54 | n=54 |  | D | T | D*T |
| C14:0 | 0.17 ± 0.03^a^ | 0.16 ± 0.02^a^ | 0.16 ± 0.02^bc^ | 0.15 ± 0.02^cd^ | 0.14 ± 0.02^de^ | 0.13 ± 0.01^e^ |  | 0.16 ± 0.03^a^ | 0.15 ± 0.02^a^ | 0.14 ± 0.02^b^ |  | **** | *** | ns |
| C16:0 | 5.52 ± 0.70^a^ | 4.95 ± 0.93^b^ | 4.92 ± 0.29^b^ | 4.25 ± 0.41^c^ | 3.43 ± 0.34^d^ | 2.92 ± 0.24^e^ |  | 4.35 ± 1.03 | 4.25 ± 1.14 | 4.41 ± 1.02 |  | **** | ns | ns |
| C17:0 | 0.05 ± 0.01^a^ | 0.05 ± 0.01^ab^ | 0.04 ± 0.00^bc^ | 0.04 ± 0.01^c^ | 0.03 ± 0.01^d^ | 0.03 ± 0.01^e^ |  | 0.04 ± 0.01 | 0.04 ± 0.01 | 0.04 ± 0.01 |  | **** | ns | ns |
| C18:0 | 0.42 ± 0.10^a^ | 0.39 ± 0.08^ab^ | 0.37 ± 0.05^b^ | 0.31 ± 0.04^c^ | 0.23 ± 0.03^d^ | 0.19 ± 0.03^e^ |  | 0.32 ± 0.10 | 0.32 ± 0.12 | 0.31 ± 0.09 |  | **** | ns | ns |
| C18:1n9c&t | 7.99 ± 1.69^a^ | 7.45 ± 1.44^a^ | 6.74 ± 0.95^b^ | 5.12 ± 0.86^c^ | 3.23 ± 0.53^d^ | 2.28 ± 0.39^e^ |  | 5.11 ± 2.19^b^ | 5.30 ± 2.35^b^ | 6.00 ± 2.56^a^ |  | **** | **** | ns |
| C18:2n6c&t | 8.55 ± 1.14^a^ | 7.86 ± 0.52^b^ | 7.17 ± 0.52^c^ | 5.83 ± 0.85^d^ | 4.18 ± 0.67^e^ | 3.24 ± 0.42^f^ |  | 6.11 ± 2.09 | 6.15 ± 2.07 | 6.14 ± 2.05 |  | **** | ns | ns |
| C18:3n3 | 0.32 ± 0.05^a^ | 0.29 ± 0.03^b^ | 0.25 ± 0.03^c^ | 0.20 ± 0.03^d^ | 0.14 ± 0.02^e^ | 0.11 ± 0.02^f^ |  | 0.21 ± 0.08 | 0.22 ± 0.08 | 0.22 ± 0.08 |  | **** | ns | ns |
| C20:0 | 0.12 ± 0.03^a^ | 0.12 ± 0.02^a^ | 0.10 ± 0.02^b^ | 0.08 ± 0.02^c^ | 0.05 ± 0.01^d^ | 0.03 ± 0.01^e^ |  | 0.07 ± 0.04^b^ | 0.08 ± 0.04^a^ | 0.09 ± 0.04^a^ |  | **** | ** | ns |
| C20:1n9 | 0.13 ± 0.03^a^ | 0.12 ± 0.03^a^ | 0.11 ± 0.02^b^ | 0.08 ± 0.02^c^ | 0.05 ± 0.02^d^ | 0.03 ± 0.01^e^ |  | 0.08 ± 0.04^c^ | 0.09 ± 0.04^b^ | 0.10 ± 0.05^a^ |  | **** | **** | ns |
| C24:0 | 0.29 ± 0.05^a^ | 0.27 ± 0.05^b^ | 0.21 ± 0.05^c^ | 0.15 ± 0.03^d^ | 0.09 ± 0.02^e^ | 0.07 ± 0.01^f^ |  | 0.16 ± 0.09^c^ | 0.18 ± 0.09^b^ | 0.20 ± 0.09^a^ |  | **** | **** | ns |
| Total | 23.55 ± 3.57^a^ | 21.65 ± 2.27^b^ | 20.06 ± 1.55^c^ | 16.20 ± 2.04^d^ | 11.57 ± 1.48^e^ | 9.03 ± 0.97^f^ |  | 16.6 ± 5.62^b^ | 16.79 ± 5.65^b^ | 17.64 ± 5.89^a^ |  | **** | * | ns |
| ∑SFA | 6.57 ± 0.87^a^ | 5.93 ± 0.93^b^ | 5.80 ± 0.34^b^ | 4.97 ± 0.48^c^ | 3.97 ± 0.39^d^ | 3.37 ± 0.27^e^ |  | 5.09 ± 1.26 | 5.03 ± 1.33 | 5.19 ± 1.25 |  | **** | ns | ns |
| ∑UFA | 16.98 ± 2.73^a^ | 15.72 ± 1.92^b^ | 14.26 ± 1.23^c^ | 11.23 ± 1.57^d^ | 7.60 ± 1.10^e^ | 5.66 ± 0.73^f^ |  | 11.51 ± 4.36^b^ | 11.76 ± 4.48^b^ | 12.46 ± 4.65^a^ |  | **** | * | ns |
| ∑MUFA | 8.12 ± 1.72^a^ | 7.57 ± 1.47^a^ | 6.85 ± 0.96^b^ | 5.20 ± 0.88^c^ | 3.28 ± 0.54^d^ | 2.31 ± 0.40^e^ |  | 5.18 ± 2.22^b^ | 5.39 ± 2.39^b^ | 6.10 ± 2.61^a^ |  | **** | **** | ns |
| ∑PUFA | 8.86 ± 1.19^a^ | 8.14 ± 0.54^b^ | 7.41 ± 0.54^c^ | 6.03 ± 0.87^d^ | 4.32 ± 0.69^e^ | 3.35 ± 0.44^f^ |  | 6.33 ± 2.17 | 6.37 ± 2.15 | 6.36 ± 2.13 |  | **** | ns | ns |
| ∑MUFA/∑PUFA | 0.91 ± 0.12^a^ | 0.92 ± 0.13^a^ | 0.93 ± 0.13^a^ | 0.87 ± 0.13^a^ | 0.77 ± 0.12^b^ | 0.70 ± 0.12^c^ |  | 0.79 ± 0.11^b^ | 0.82 ± 0.14^b^ | 0.94 ± 0.16^a^ |  | **** | **** | ns |
| ∑Long(13-21) | 23.26 ± 3.53^a^ | 21.38 ± 2.23^b^ | 19.85 ± 1.52^c^ | 16.06 ± 2.01^d^ | 11.48 ± 1.46^e^ | 8.96 ± 0.97^f^ |  | 16.44 ± 5.54^b^ | 16.61 ± 5.56^b^ | 17.45 ± 5.80^a^ |  | **** | * | ns |
| ∑Very long(>22) | 0.29 ± 0.05^a^ | 0.27 ± 0.05^b^ | 0.21 ± 0.05^c^ | 0.15 ± 0.03^d^ | 0.09 ± 0.02^e^ | 0.07 ± 0.01^f^ |  | 0.16 ± 0.09^c^ | 0.18 ± 0.09^b^ | 0.20 ± 0.09^a^ |  | **** | **** | ns |
| ∑n-6 PUFA | 8.55 ± 1.14^a^ | 7.86 ± 0.52^b^ | 7.17 ± 0.52^c^ | 5.83 ± 0.85^d^ | 4.18 ± 0.67^e^ | 3.24 ± 0.42^f^ |  | 6.11 ± 2.09 | 6.15 ± 2.07 | 6.14 ± 2.05 |  | **** | ns | ns |
| ∑n-3 PUFA | 0.32 ± 0.05^a^ | 0.29 ± 0.03^b^ | 0.25 ± 0.03^c^ | 0.20 ± 0.03^d^ | 0.14 ± 0.02^e^ | 0.11 ± 0.02^f^ |  | 0.21 ± 0.08 | 0.22 ± 0.08 | 0.22 ± 0.08 |  | **** | ns | ns |
| ∑n-6/∑n-3 | 26.94 ± 1.58^b^ | 27.55 ± 1.93^b^ | 28.92 ± 2.11^a^ | 29.18 ± 1.71^a^ | 29.90 ± 2.19^a^ | 29.56 ± 2.79^a^ |  | 29.26 ± 2.93^a^ | 28.73 ± 1.71^ab^ | 28.04 ± 2.02^b^ |  | **** | ** | ns |

^a-f^ Values with different superscripts differ significantly with respect to the degree of milling or rice type (p<0.05). ns: Non-significant, * p < 0.05, ** p < 0.01, *** p < 0.001, **** p < 0.0001, ∑SFA: Sum of saturated fatty acids, ∑UFA: Sum of unsaturated fatty acids, ∑MUFA: Sum of monounsaturated fatty acids, ∑PUFA: Sum of polyunsaturated fatty acids, ∑MUFA/∑PUFA: Ratio of monounsaturated fatty acids and polyunsaturated fatty acids, ∑Long: Sum of long-chain fatty acids with 13-21 carbons, ∑Very long: Sum of long-chain fatty acids with more than 22 carbons, ∑n-3 PUFA: Sum of omega-3 fatty acids, ∑n-6 PUFA: Sum of omega-6 fatty acids, ∑n-3/∑n-6; Ratio of omega-3 fatty acids and omega-6 fatty acids, DOM: Degree of milling, OR: Organic rice, PFR: Pesticide-free rice, CR: Conventional rice

Table S4. Dependence of vitamin E composition and content in rice samples on the degree of milling (DOM) and rice type (µg·g^-1^ on dry weight basis)

| Vitamin E | Degree of milling (D) | | | | | |  | Rice type (T) | | |  | *p*-Value | | |
| --- | --- | --- | --- | --- | --- | --- | --- | --- | --- | --- | --- | --- | --- | --- |
|  | Brown rice | 5 DOM | 7 DOM | 9 DOM | 11 DOM | 13 DOM |  | OR | PFR | CR |  | Main factor | | Interaction |
|  | n=27 | n=27 | n=27 | n=27 | n=27 | n=27 |  | n=54 | n=54 | n=54 |  | D | T | D*T |
| γT | 1.56 ± 0.30^a^ | 1.49 ± 0.19^ab^ | 1.33 ± 0.22^b^ | 1.13 ± 0.14^c^ | 1.02 ± 0.09^cd^ | 0.89 ± 0.02^d^ |  | 1.22 ± 0.29^b^ | 1.36 ± 0.26^a^ | 1.39 ± 0.28^a^ |  | **** | **** | Ns |
| αT | 7.33 ± 1.05^a^ | 7.07 ± 0.89^a^ | 6.35 ± 0.85^b^ | 4.61 ± 0.81^c^ | 2.74 ± 0.85^d^ | 1.81 ± 0.69^e^ |  | 4.32 ± 2.31^c^ | 5.64 ± 2.22^a^ | 5.00 ± 2.20^b^ |  | **** | **** | Ns |
| γT_3_ | 5.58 ± 0.94^a^ | 5.43 ± 0.87^a^ | 4.98 ± 0.84^b^ | 4.25 ± 0.61^c^ | 3.56 ± 0.53^d^ | 3.12 ± 0.45^e^ |  | 4.15 ± 1.31^b^ | 4.67 ± 1.14^a^ | 4.64 ± 0.99^a^ |  | **** | *** | Ns |
| αT_3_ | 2.95 ± 0.72^a^ | 2.85 ± 0.67^a^ | 2.49 ± 0.57^b^ | 1.95 ± 0.37^c^ | 1.50 ± 0.26^d^ | 1.23 ± 0.16^e^ |  | 1.89 ± 0.76^c^ | 2.42 ± 0.85^a^ | 2.17 ± 0.77^b^ |  | *** | **** | Ns |
| Total | 17.42 ± 2.72^a^ | 16.84 ± 2.45^a^ | 15.15 ± 2.23^b^ | 11.82 ± 1.63^c^ | 8.25 ± 1.91^d^ | 6.26 ± 1.22^e^ |  | 11.10 ± 4.74^c^ | 13.87 ± 4.62^a^ | 12.90 ± 4.45^b^ |  | **** | **** | Ns |

^a-e^ Values with different superscripts differ significantly with respect to the degree of milling or rice type (p<0.05). ns: Non-significant, *p < 0.05, **p < 0.01, ***p < 0.001, ****p < 0.0001, γT: γ-Tocopherol, αT: α-Tocopherol, γT3: γ-Tocotrienol, αT3: α-Tocotrienol, DOM: Degree of milling, OR: Organic rice, PFR: Pesticide-free rice, CR: Conventional rice

Table S5. Dependence of phenolic composition and content of rice samples on the degree of milling (DOM) and rice type (µg·g^-1^ on dry weight basis)

| Phenolic compounds | Degree of milling (D) | | | | | |  | Rice type (T) | | |  | *p*-Value | | |
| --- | --- | --- | --- | --- | --- | --- | --- | --- | --- | --- | --- | --- | --- | --- |
|  | Brown rice | 5 DOM | 7 DOM | 9 DOM | 11 DOM | 13 DOM |  | OR | PFR | CR |  | Main factor | | Interaction |
|  | n=27 | n=27 | n=27 | n=27 | n=27 | n=27 |  | n=54 | n=54 | n=54 |  | D | T | D*T |
| PCA | 0.29 ± 0.05 | 0.29 ± 0.03 | 0.28 ± 0.03 | 0.30 ± 0.07 | 0.27 ± 0.02 | 0.29 ± 0.05 |  | 0.30 ± 0.05^a^ | 0.28 ± 0.05^b^ | 0.28 ± 0.04^b^ |  | ns | * | ns |
| *p*HBA | 0.51 ± 0.14 | 0.51 ± 0.15 | 0.51 ± 0.15 | 0.49 ± 0.17 | 0.44 ± 0.15 | 0.43 ± 0.17 |  | 0.51 ± 0.17^a^ | 0.51 ± 0.19^a^ | 0.43 ± 0.08^b^ |  | ns | ** | ns |
| GTA | 0.18 ± 0.03 | 0.18 ± 0.03 | 0.18 ± 0.03 | 0.18 ± 0.03 | 0.19 ± 0.04 | 0.19 ± 0.03 |  | 0.20 ± 0.04^a^ | 0.19 ± 0.02^b^ | 0.16 ± 0.01^c^ |  | ns | **** | ns |
| *p*CMA | 0.77 ± 0.14^a^ | 0.77 ± 0.13^a^ | 0.76 ± 0.13^a^ | 0.66 ± 0.13^b^ | 0.58 ± 0.13^c^ | 0.51 ± 0.12^c^ |  | 0.68 ± 0.21 | 0.69 ± 0.16 | 0.65 ± 0.12 |  | **** | ns | ns |
| CA | 0.16 ± 0.06 | 0.16 ± 0.06 | 0.17 ± 0.06 | 0.16 ± 0.07 | 0.16 ± 0.07 | 0.16 ± 0.06 |  | 0.19 ± 0.09^a^ | 0.16 ± 0.03^b^ | 0.13 ± 0.03^c^ |  | ns | **** | ns |
| SA | 0.49 ± 0.13^ab^ | 0.52 ± 0.14^a^ | 0.53 ± 0.13^a^ | 0.44 ± 0.12^bc^ | 0.38 ± 0.13^cd^ | 0.33 ± 0.13^d^ |  | 0.45 ± 0.20 | 0.45 ± 0.13 | 0.45 ± 0.11 |  | **** | ns | ns |
| FA | 1.86 ± 0.36^a^ | 1.89 ± 0.36^a^ | 1.90 ± 0.29^a^ | 1.79 ± 0.25^a^ | 1.60 ± 0.24^b^ | 1.50 ± 0.28^b^ |  | 1.76 ± 0.24 | 1.75 ± 0.27 | 1.77 ± 0.45 |  | **** | ns | ns |
| Total | 4.26 ± 0.46^ab^ | 4.31 ± 0.50^a^ | 4.32 ± 0.40^a^ | 4.03 ± 0.42^b^ | 3.63 ± 0.40^c^ | 3.40 ± 0.44^c^ |  | 4.08 ± 0.48^a^ | 4.02 ± 0.51^ab^ | 3.87 ± 0.67^b^ |  | **** | * | ns |

^a-d^ Values with different superscripts differ significantly with respect to the degree of milling or rice type (p<0.05). ns: Non-significant, *p < 0.05, **p < 0.01, ***p < 0.001, ****p < 0.0001, PCA: Protocatechuic acid, pHBA: *p*-Hydroxybenzoic acid, GTA: Gentisic acid, pCMA: *p*-Coumaric acid, CA: Caffeic acid, SA: Salicylic acid, FA: Ferulic acid, DOM: Degree of milling, OR: Organic rice, PFR: Pesticide-free rice, CR: Conventional rice

Table S6. Fatty acid composition and content (mg·g^-1^ on dry weight basis) in rice types with the same degree of milling (DOM)

| Fatty acid | Degree of milling | | | | | | | | | | | | | | | | | | | | | | |
| --- | --- | --- | --- | --- | --- | --- | --- | --- | --- | --- | --- | --- | --- | --- | --- | --- | --- | --- | --- | --- | --- | --- | --- |
|  | Brown rice | | |  | 5 DOM | | |  | 7 DOM | | |  | 9 DOM | | |  | 11 DOM | | |  | 13 DOM | | |
|  | OR | PFR | CR |  | OR | PFR | CR |  | OR | PFR | CR |  | OR | PFR | CR |  | OR | PFR | CR |  | OR | PFR | CR |
|  | n=9 | n=9 | n=9 |  | n=9 | n=9 | n=9 |  | n=9 | n=9 | n=9 |  | n=9 | n=9 | n=9 |  | n=9 | n=9 | n=9 |  | n=9 | n=9 | n=9 |
| C14:0 | 0.17 | 0.18 | 0.15 |  | 0.16 | 0.16 | 0.15 |  | 0.16 | 0.16 | 0.15 |  | 0.15 | 0.15 | 0.14 |  | 0.15^a^ | 0.14^ab^ | 0.13^b^ |  | 0.14 | 0.13 | 0.12 |
| C16:0 | 5.55 | 5.57 | 5.44 |  | 5.07 | 4.48 | 5.29 |  | 4.86^ab^ | 4.81^b^ | 5.09^a^ |  | 4.27 | 4.29 | 4.21 |  | 3.36 | 3.46 | 3.48 |  | 2.96 | 2.88 | 2.93 |
| C17:0 | 0.05 | 0.05 | 0.05 |  | 0.05 | 0.04 | 0.05 |  | 0.04 | 0.04 | 0.04 |  | 0.04 | 0.04 | 0.04 |  | 0.04^a^ | 0.04^a^ | 0.03^b^ |  | 0.03 | 0.03 | 0.03 |
| C18:0 | 0.42 | 0.43 | 0.40 |  | 0.39 | 0.39 | 0.39 |  | 0.36 | 0.36 | 0.37 |  | 0.31 | 0.32 | 0.29 |  | 0.22 | 0.23 | 0.23 |  | 0.19 | 0.19 | 0.18 |
| C18:1n9c&t | 7.51 | 7.94 | 8.53 |  | 6.94 | 7.16 | 8.25 |  | 6.28^b^ | 6.31^b^ | 7.62^a^ |  | 4.91 | 5.03 | 5.42 |  | 2.84^b^ | 3.23^ab^ | 3.62^a^ |  | 2.17^b^ | 2.14^b^ | 2.54^a^ |
| C18:2n6c&t | 8.60 | 8.67 | 8.36 |  | 7.85 | 7.72 | 8.00 |  | 7.08 | 7.04 | 7.37 |  | 5.87 | 5.98 | 5.64 |  | 4.01 | 4.30 | 4.23 |  | 3.28 | 3.21 | 3.22 |
| C18:3n3 | 0.32 | 0.32 | 0.32 |  | 0.28^ab^ | 0.27^b^ | 0.30^a^ |  | 0.24^b^ | 0.24^ab^ | 0.27^a^ |  | 0.20 | 0.20 | 0.20 |  | 0.13 | 0.14 | 0.14 |  | 0.11 | 0.11 | 0.11 |
| C20:0 | 0.12 | 0.13 | 0.12 |  | 0.11 | 0.12 | 0.12 |  | 0.09^b^ | 0.10^a^ | 0.11^a^ |  | 0.07 | 0.08 | 0.08 |  | 0.04^b^ | 0.05^a^ | 0.05^a^ |  | 0.03 | 0.03 | 0.03 |
| C20:1n9 | 0.12 | 0.13 | 0.14 |  | 0.11^b^ | 0.12^ab^ | 0.14^a^ |  | 0.09^c^ | 0.11^b^ | 0.13^a^ |  | 0.07^b^ | 0.09^ab^ | 0.09^a^ |  | 0.04^b^ | 0.06^a^ | 0.06^a^ |  | 0.03 | 0.03 | 0.03 |
| C24:0 | 0.27 | 0.29 | 0.30 |  | 0.25^b^ | 0.25^b^ | 0.30^a^ |  | 0.16^c^ | 0.20^b^ | 0.25^a^ |  | 0.13 | 0.15 | 0.16 |  | 0.07^b^ | 0.10^a^ | 0.11^a^ |  | 0.06^b^ | 0.07^ab^ | 0.08^a^ |
| Total | 23.11 | 23.71 | 23.83 |  | 21.23^ab^ | 20.73^b^ | 22.99^a^ |  | 19.37^b^ | 19.39^b^ | 21.42^a^ |  | 16.03 | 16.32 | 16.26 |  | 10.89 | 11.75 | 12.08 |  | 8.99 | 8.82 | 9.28 |
| ∑SFA | 6.58 | 6.65 | 6.47 |  | 6.04 | 5.46 | 6.30 |  | 5.68^b^ | 5.69^b^ | 6.02^a^ |  | 4.98 | 5.02 | 4.91 |  | 3.87 | 4.02 | 4.02 |  | 3.41 | 3.33 | 3.38 |
| ∑UFA | 16.54 | 17.06 | 17.36 |  | 15.19 | 15.28 | 16.69 |  | 13.69^b^ | 13.70^b^ | 15.39^a^ |  | 11.05 | 11.30 | 11.35 |  | 7.02^b^ | 7.73^ab^ | 8.05^a^ |  | 5.58 | 5.49 | 5.91 |
| ∑MUFA | 7.62 | 8.06 | 8.68 |  | 7.05 | 7.28 | 8.38 |  | 6.37^b^ | 6.42^b^ | 7.76^a^ |  | 4.98 | 5.12 | 5.51 |  | 2.88^b^ | 3.29^ab^ | 3.68^a^ |  | 2.20^b^ | 2.16^b^ | 2.58^a^ |
| ∑PUFA | 8.92 | 8.99 | 8.68 |  | 8.14 | 7.99 | 8.30 |  | 7.32 | 7.28 | 7.64 |  | 6.07 | 6.18 | 5.84 |  | 4.14 | 4.44 | 4.38 |  | 3.39 | 3.32 | 3.33 |
| ∑MUFA/∑PUFA | 0.86^b^ | 0.89^ab^ | 0.99^a^ |  | 0.86^b^ | 0.91^ab^ | 1.00^a^ |  | 0.87^b^ | 0.88^b^ | 1.03^a^ |  | 0.82^b^ | 0.83^b^ | 0.95^a^ |  | 0.7^b^ | 0.75^b^ | 0.86^a^ |  | 0.65^b^ | 0.66^b^ | 0.78^a^ |
| ∑Long(13-21) | 22.85 | 23.41 | 23.53 |  | 20.98^ab^ | 20.48^b^ | 22.69^a^ |  | 19.2^b^ | 19.19^b^ | 21.17^a^ |  | 15.89 | 16.18 | 16.10 |  | 10.82 | 11.65 | 11.97 |  | 8.93 | 8.75 | 9.21 |
| ∑Very long(>22) | 0.27 | 0.29 | 0.30 |  | 0.25^b^ | 0.25^b^ | 0.30^a^ |  | 0.16^c^ | 0.20^b^ | 0.25^a^ |  | 0.13 | 0.15 | 0.16 |  | 0.07^b^ | 0.10^a^ | 0.11^a^ |  | 0.06^b^ | 0.07^ab^ | 0.08^a^ |
| ∑n-6 PUFA | 8.60 | 8.67 | 8.36 |  | 7.85 | 7.72 | 8.00 |  | 7.08 | 7.04 | 7.37 |  | 5.87 | 5.98 | 5.64 |  | 4.01 | 4.30 | 4.23 |  | 3.28 | 3.21 | 3.22 |
| ∑n-3 PUFA | 0.32 | 0.32 | 0.32 |  | 0.28^ab^ | 0.27b | 0.30^b^ |  | 0.24^b^ | 0.24^ab^ | 0.27^a^ |  | 0.20 | 0.20 | 0.20 |  | 0.13 | 0.14 | 0.14 |  | 0.11 | 0.11 | 0.11 |
| ∑n-6/∑n-3 | 27.38 | 27.16 | 26.29 |  | 27.74^ab^ | 28.58^a^ | 26.34^b^ |  | 29.95^a^ | 29.03^ab^ | 27.8^b^ |  | 29.04 | 29.64 | 28.88 |  | 30.38 | 29.94 | 29.39 |  | 31.06^a^ | 28.05^b^ | 29.58^ab^ |

^a-c^ Values with different superscripts differ significantly with respect to the rice type (p<0.05). ∑SFA: Sum of saturated fatty acids, ∑UFA: Sum of unsaturated fatty acids, ∑MUFA: Sum of monounsaturated fatty acids, ∑PUFA: Sum of polyunsaturated fatty acids, ∑MUFA/∑PUFA: Ratio of monounsaturated fatty acids and polyunsaturated fatty acids, ∑Long: Sum of long-chain fatty acids with 13-21 carbons, ∑Very long: Sum of long-chain fatty acids with more than 22 carbons, ∑n-3 PUFA: Sum of omega-3 fatty acids, ∑n-6 PUFA: Sum of omega-6 fatty acids, ∑n-3/∑n-6; Ratio of omega-3 fatty acids and omega-6 fatty acids, DOM: Degree of milling, OR: Organic rice, PFR: Pesticide-free rice, CR: Conventional rice

Table S7. Vitamin E composition and content (µg·g^-1^ on dry weight basis) in rice types with the same degree of milling (DOM).

| Vitamin E | Degree of milling | | | | | | | | | | | | | | | | | | | | | | |  |
| --- | --- | --- | --- | --- | --- | --- | --- | --- | --- | --- | --- | --- | --- | --- | --- | --- | --- | --- | --- | --- | --- | --- | --- | --- |
|  | Brown rice | | |  | 5 DOM | | |  | 7 DOM | | |  | 9 DOM | | |  | 11 DOM | | |  | 13 DOM | | | |
|  | OR | PFR | CR |  | OR | PFR | CR |  | OR | PFR | CR |  | OR | PFR | CR |  | OR | PFR | CR |  | OR | PFR | CR | |
|  | n=9 | n=9 | n=9 |  | n=9 | n=9 | n=9 |  | n=9 | n=9 | n=9 |  | n=9 | n=9 | n=9 |  | n=9 | n=9 | n=9 |  | n=9 | n=9 | n=9 | |
| γT | 1.46 | 1.58 | 1.63 |  | 1.27^b^ | 1.61^a^ | 1.57^a^ |  | 1.13^b^ | 1.44^a^ | 1.43^a^ |  | 0.94^c^ | 1.15^b^ | 1.23^a^ |  | nd | 1.02 | 1.02 |  | nd | nd | 0.89 | |
| αT | 6.51^b^ | 7.97^a^ | 7.51^a^ |  | 6.30^c^ | 7.79^a^ | 7.12^b^ |  | 5.96^b^ | 7.01^a^ | 6.08^b^ |  | 4.17^b^ | 5.12^a^ | 4.54^ab^ |  | 1.83^b^ | 3.44^a^ | 2.96^a^ |  | 1.12^c^ | 2.52^a^ | 1.78^b^ | |
| γT_3_ | 5.22 | 5.79 | 5.73 |  | 5.01 | 5.75 | 5.54 |  | 4.66 | 5.17 | 5.12 |  | 4.13 | 4.23 | 4.40 |  | 3.14^b^ | 3.81^a^ | 3.72^a^ |  | 2.72^b^ | 3.29^a^ | 3.35^a^ | |
| αT_3_ | 2.42^b^ | 3.39^a^ | 3.04^a^ |  | 2.36^b^ | 3.26^a^ | 2.92^ab^ |  | 2.20^b^ | 2.82^a^ | 2.43^ab^ |  | 1.98 | 1.99 | 1.88 |  | 1.25^b^ | 1.71^a^ | 1.54^a^ |  | 1.11^b^ | 1.37^a^ | 1.21^b^ | |
| Total | 15.61^b^ | 18.73^a^ | 17.91^ab^ |  | 14.94^b^ | 18.42^a^ | 17.15^a^ |  | 13.95^b^ | 16.44^a^ | 15.07^ab^ |  | 10.91^b^ | 12.49^a^ | 12.05^ab^ |  | 6.22^c^ | 9.97^a^ | 8.55^b^ |  | 4.95^b^ | 7.17^a^ | 6.64^a^ | |

^a-c^ Values with different superscripts differ significantly with respect to the rice type (p<0.05). nd: Non-detected, γT: γ-Tocopherol, αT: α-Tocopherol, γT3: γ-Tocotrienol, αT3: α-Tocotrienol, DOM: Degree of milling, OR: Organic rice, PFR: Pesticide-free rice, CR: Conventional rice

Table S8. Phenolic composition and content (µg·g^-1^ on dry weight basis) in rice types with the same degree of milling (DOM)

| Phenolic compound | Degree of milling | | | | | | | | | | | | | | | | | | | | | | |
| --- | --- | --- | --- | --- | --- | --- | --- | --- | --- | --- | --- | --- | --- | --- | --- | --- | --- | --- | --- | --- | --- | --- | --- |
|  | Brown rice | | |  | 5 DOM | | |  | 7 DOM | | |  | 9 DOM | | |  | 11 DOM | | |  | 13 DOM | | |
|  | OR | PFR | CR |  | OR | PFR | CR |  | OR | PFR | CR |  | OR | PFR | CR |  | OR | PFR | CR |  | OR | PFR | CR |
|  | n=9 | n=9 | n=9 |  | n=9 | n=9 | n=9 |  | n=9 | n=9 | n=9 |  | n=9 | n=9 | n=9 |  | n=9 | n=9 | n=9 |  | n=9 | n=9 | n=9 |
| PCA | 0.30 | 0.27 | 0.29 |  | 0.31^a^ | 0.27^b^ | 0.29^ab^ |  | 0.28 | 0.28 | 0.28 |  | 0.30 | 0.30 | 0.28 |  | 0.28^a^ | 0.27^ab^ | 0.26^b^ |  | 0.32^a^ | 0.28^ab^ | 0.26^b^ |
| *p*HBA | 0.52 | 0.55 | 0.47 |  | 0.55 | 0.52 | 0.47 |  | 0.53 | 0.52 | 0.48 |  | 0.52 | 0.53 | 0.42 |  | 0.49 | 0.47 | 0.38 |  | 0.46 | 0.48 | 0.36 |
| GTA | 0.19^a^ | 0.18^ab^ | 0.16^b^ |  | 0.20^a^ | 0.18^ab^ | 0.16^b^ |  | 0.19^a^ | 0.19^a^ | 0.16^b^ |  | 0.20^a^ | 0.19^ab^ | 0.16^b^ |  | 0.20^a^ | 0.19^ab^ | 0.17^b^ |  | 0.20^a^ | 0.19^ab^ | 0.17^b^ |
| *p*CMA | 0.75 | 0.83 | 0.74 |  | 0.80 | 0.75 | 0.75 |  | 0.77 | 0.77 | 0.74 |  | 0.66 | 0.69 | 0.63 |  | 0.60 | 0.57 | 0.57 |  | 0.53 | 0.50 | 0.50 |
| CA | 0.18 | 0.16 | 0.13 |  | 0.19^a^ | 0.16^ab^ | 0.13^b^ |  | 0.20^a^ | 0.17^ab^ | 0.13^b^ |  | 0.20^a^ | 0.16^ab^ | 0.13^b^ |  | 0.20^a^ | 0.17^ab^ | 0.13^b^ |  | 0.19 | 0.15 | 0.13 |
| SA | 0.46 | 0.49 | 0.53 |  | 0.53 | 0.50 | 0.52 |  | 0.52 | 0.53 | 0.54 |  | 0.44 | 0.46 | 0.44 |  | 0.39 | 0.38 | 0.38 |  | 0.35 | 0.34 | 0.30 |
| FA | 1.83 | 1.85 | 1.91 |  | 1.87 | 1.86 | 1.93 |  | 1.87 | 1.95 | 1.88 |  | 1.82 | 1.78 | 1.78 |  | 1.59 | 1.58 | 1.63 |  | 1.55 | 1.47 | 1.49 |
| Total | 4.23 | 4.33 | 4.22 |  | 4.45 | 4.24 | 4.25 |  | 4.35 | 4.41 | 4.20 |  | 4.13 | 4.11 | 3.84 |  | 3.75 | 3.63 | 3.52 |  | 3.58 | 3.42 | 3.21 |

^a-c^ Values with different superscripts differ significantly with respect to the rice type (p<0.05). PCA: Protocatechuic acid, pHBA: *p*-Hydroxybenzoic acid, GTA: Gentisic acid, *p*CMA: *p*-Coumaric acid, CA: Caffeic acid, SA: Salicylic acid, FA: Ferulic acid, DOM: Degree of milling, OR: Organic rice, PFR: Pesticide-free rice, CR: Conventional rice

Table S9. Classification and cross-validated results^a,b^ for rice DOM identification (six levels) using the SDA method

|  |  | DOM level | Predicted group membership | | | | | | Total |
| --- | --- | --- | --- | --- | --- | --- | --- | --- | --- |
|  |  |  | BR | 5 DOM | 7 DOM | 9 DOM | 11 DOM | 13 DOM |  |
| Original | Count | BR | 18 | 8 | 1 | 0 | 0 | 0 | 27 |
|  |  | 5 DOM | 7 | 17 | 3 | 0 | 0 | 0 | 27 |
|  |  | 7 DOM | 0 | 5 | 22 | 0 | 0 | 0 | 27 |
|  |  | 9 DOM | 0 | 0 | 2 | 24 | 1 | 0 | 27 |
|  |  | 11 DOM | 0 | 0 | 0 | 0 | 23 | 4 | 27 |
|  |  | 13 DOM | 0 | 0 | 0 | 0 | 3 | 24 | 27 |
|  | % | 1 | 66.7 | 29.6 | 3.7 | 0 | 0 | 0 | 100.0 |
|  |  | 2 | 25.9 | 63.0 | 11.1 | 0 | 0 | 0 | 100.0 |
|  |  | 3 | 0 | 18.5 | 81.5 | 0 | 0 | 0 | 100.0 |
|  |  | 4 | 0 | 0 | 7.4 | 88.9 | 3.7 | 0 | 100.0 |
|  |  | 5 | 0 | 0 | 0 | 0 | 85.2 | 14.8 | 100.0 |
|  |  | 6 | 0 | 0 | 0 | 0 | 11.1 | 88.9 | 100.0 |
| Cross-validated^c^ | Count | 1 | 16 | 10 | 1 | 0 | 0 | 0 | 27 |
|  |  | 2 | 8 | 15 | 4 | 0 | 0 | 0 | 27 |
|  |  | 3 | 0 | 5 | 22 | 0 | 0 | 0 | 27 |
|  |  | 4 | 0 | 0 | 3 | 23 | 1 | 0 | 27 |
|  |  | 5 | 0 | 0 | 0 | 0 | 20 | 7 | 27 |
|  |  | 6 | 0 | 0 | 0 | 0 | 3 | 24 | 27 |
|  | % | 1 | 59.3 | 37.0 | 3.7 | 0 | 0 | 0 | 100.0 |
|  |  | 2 | 29.6 | 55.6 | 14.8 | 0 | 0 | 0 | 100.0 |
|  |  | 3 | 0 | 18.5 | 81.5 | 0 | 0 | 0 | 100.0 |
|  |  | 4 | 0 | 0 | 11.1 | 85.2 | 3.7 | 0 | 100.0 |
|  |  | 5 | 0 | 0 | 0 | .0 | 74.1 | 25.9 | 100.0 |
|  |  | 6 | 0 | 0 | 0 | .0 | 11.1 | 88.9 | 100.0 |

^a^ 79.0% of original grouped cases correctly classified

^b^ 74.1% of cross-validated grouped cases correctly classified

^c^ Cross validation is performed only for cases included in the analysis. During cross validation, each case is classified by functions derived from all cases other than that case.


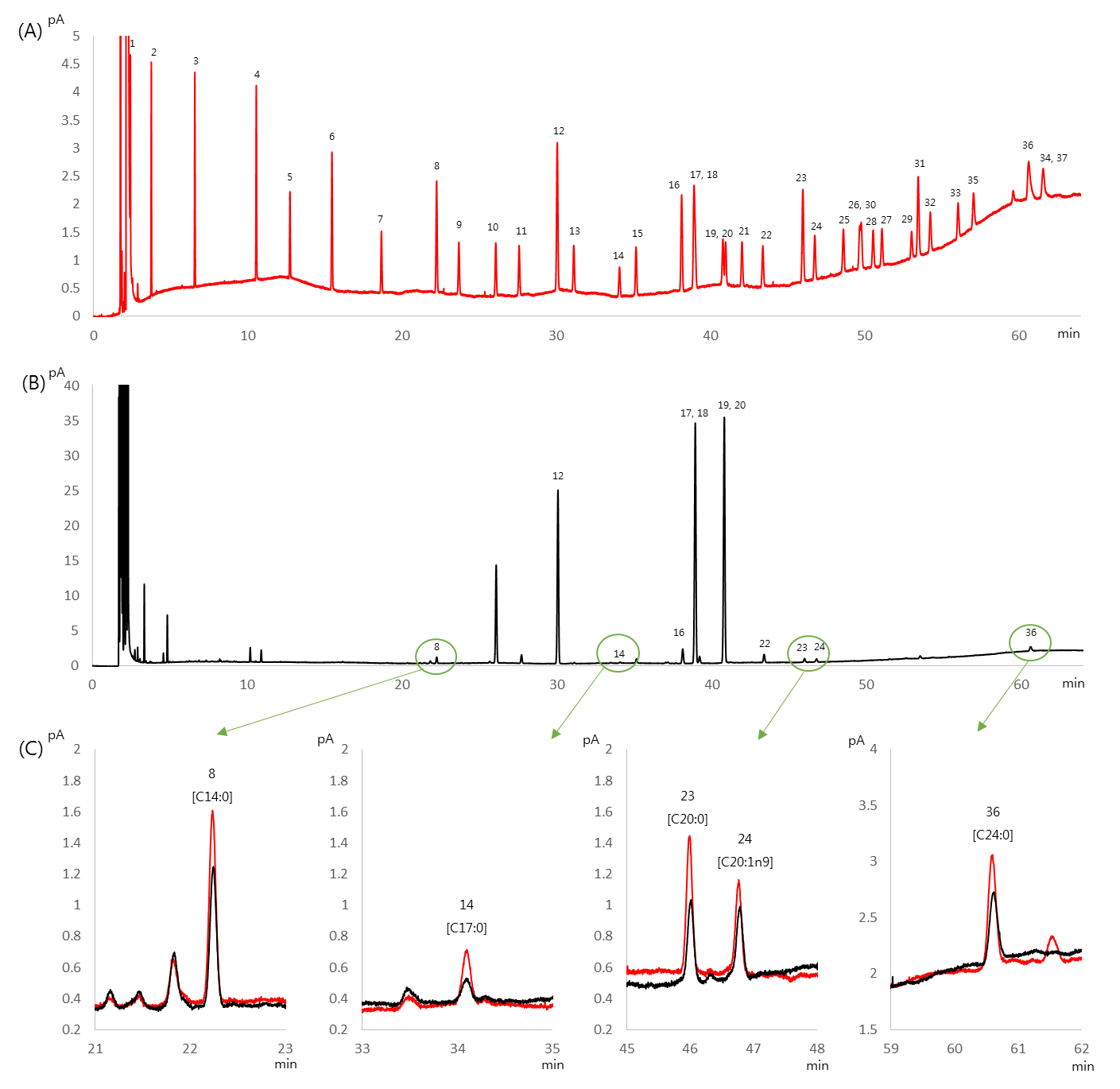


Figure S1. Representative gas chromatography-flame ionization detector (GC-FID) chromatograms: (A) 37 FAME standards mixture, (B) OR (a brown rice type), (C) The same OR chromatogram spiked with fatty acid standards (C14:0, C17:0, C20:0, C20:1, C24:0) 1; C4:0, 2; C6:0, 3; C8:0, 4; C10:0, 5; C11:0, 6; C12:0, 7; C13:0, 8; C14:0, 9; C14:1, 10; C15:0, 11; C15:1, 12;C16:0, 13; C16:1, 14; C17:0, 15; C17:1, 16; C18:0, 17+18; C18:1n9 t and c, 19, 20; C18:2n6 t and c, 21; C18:3n6, 22; C18:3n3, 23; C20:0, 24; C20:1n9, 25; C20:2, 26+30; C20:3n6 and C21:0, 27; C20:3n3, 28; C20:4n6, 29; C20:5n3, 31; C22:0, 32; C22:1n9, 33; C22:2, 35: C23:0, 36; C24:0, 34+37; C22:6n3 and C24:1n9


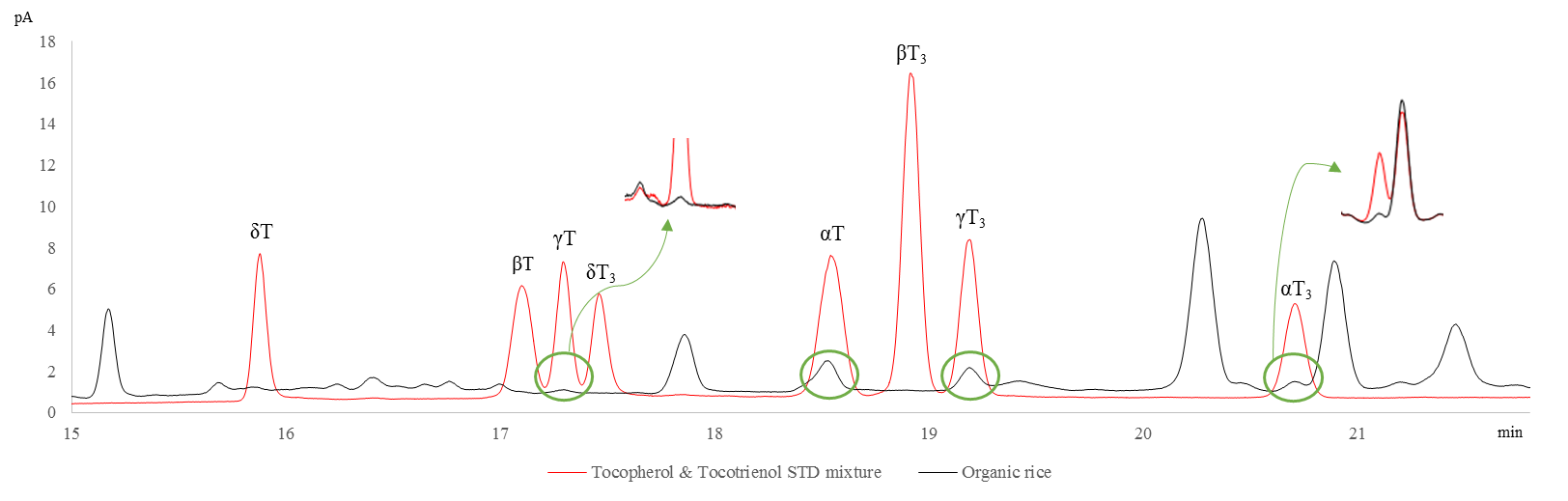


Figure S2. Representative GC-FID chromatogram of the mixture of eight tocopherol and tocotrienol standards (red solid line) and the OR with brown rice type (black solid line). α, β, γ, δT; α-, β-, γ-, δ-Tocopherol, α, β, γ, δT3; α-, β-, γ-, δ-Tocotrienol.


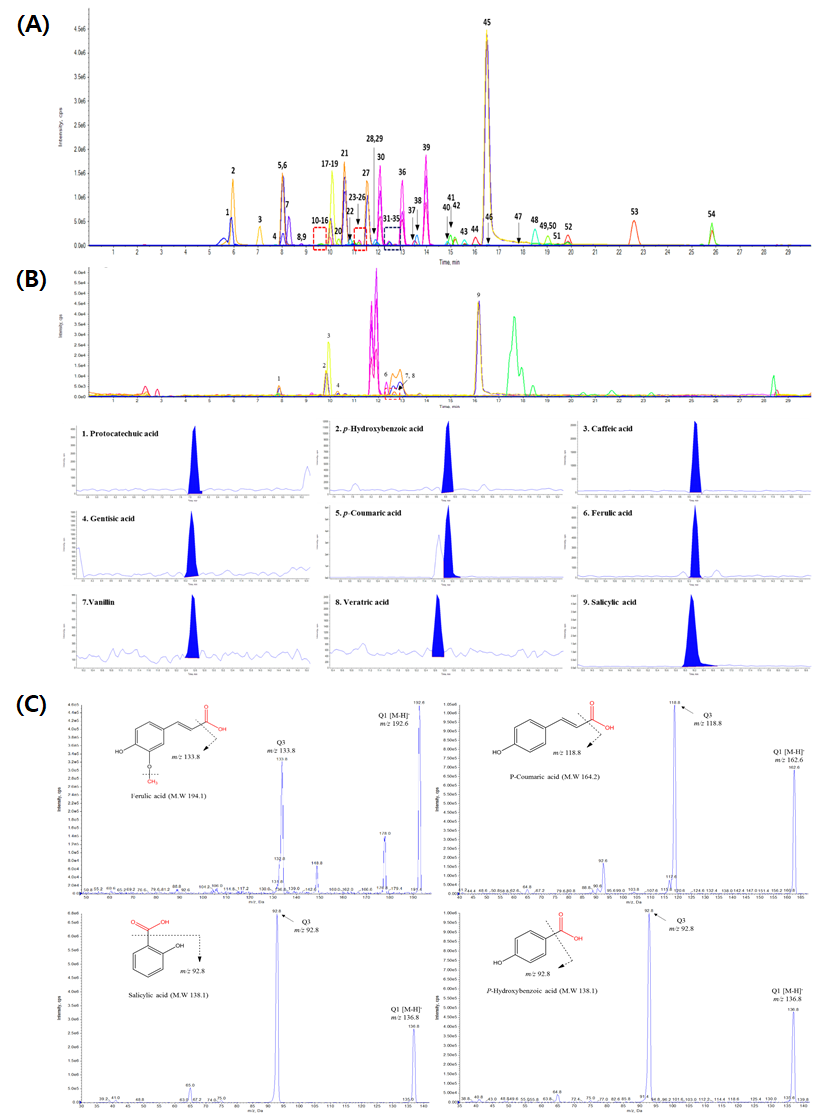


Figure S3. Representative multiple reaction monitoring (MRM) ion chromatogram of standards of 54 selected phenolic compounds; (A), the total and extracted ion chromatograms of brown rice and organic rice (B), and MS/MS spectra and fragmentation of the representative phenolic standards (C) 1. Gallic acid; 2. 5-Sulfosalicylic acid; 3. Homogentisic acid; 4. Peonidin 3-O-glucoside chloride; 5. Protocatechuic acid; 6. Delphinidin chloride; 7. Chlorogenic acid; 8. Catechin; 9. Cyanidin chloride; 10. Daidzin; 11. Glycitin; 12. Orientin; 13. Pelargonidin chloride; 14.Malvidin chloride; 15.Peonidin chloride; 16.Rutin; 17. p-Hydroxybenzoic acid; 18. Caffeic acid; 19. Syringic acid; 20. Vitexin; 21. Gentisic acid; 22. Polydatin; 23. Malonyl Glycitin; 24. Malonyl Daidzin; 25. Genistin; 26. Naringin; 27. β-Resorcylic acid; 28. Acetyl Daidzin; 29. Acetyl Glycitin; 30. p-Coumaric acid; 31. Vanillic acid; 32. Ferulic acid; 33. Malonyl Genistin; 34. Vanillin; 35. Veratric acid; 36. m-Coumaric acid; 37. Myricetin; 38. Acetyl Genistin; 39. o-Coumaric acid; 40. trans-Resveratrol; 41. Daidzein; 42. Glycitein; 43. Luteolin; 44. Quercetin; 45. Salicylic acid; 46. cis-Resveratrol; 47. trans-Cinnamic acid; 48. Apigenin; 49. Naringenin; 50. Genistein; 51. Kaempferol; 52. Hesperetin; 53. Formononetin; 54. Biochanin A.


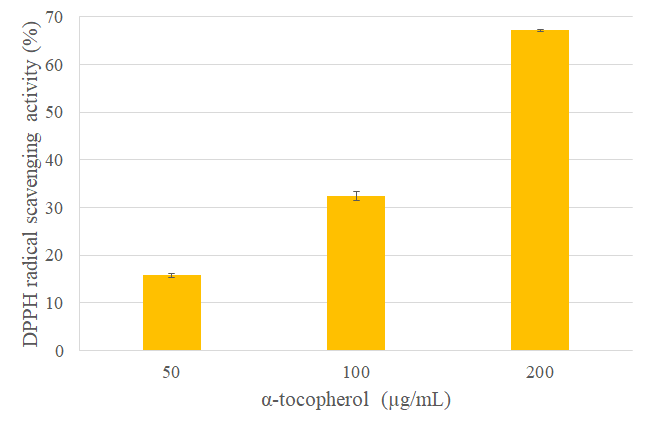


Figure S4. DPPH free radical scavenging activity according to three concentration levels of α-tocopherol.
